# Determinants of cervical cancer screening intention among reproductive age women in Ethiopia: A systematic review and meta-analysis

**DOI:** 10.1371/journal.pone.0312449

**Published:** 2024-10-31

**Authors:** Begetayinoral Kussia Lahole, Melkamu Woldamlak, Wondafrash Kussia

**Affiliations:** 1 Department of Midwifery, College of Medicine and Health Sciences, Arba Minch University, Arbaminch, Ethiopia; 2 Department of Radiology Technology, College of Medicine and Health Sciences, Arba Minch University, Arbaminch, Ethiopia; 3 School of Anesthesia, College of Health Sciences and Medicine, Wolaita Sodo University, Wolaita Sodo, Ethiopia; UFRN: Universidade Federal do Rio Grande do Norte, BRAZIL

## Abstract

**Introduction:**

Cervical cancer is a leading cause of cancer-related mortality in Ethiopia, despite being preventable. Screening programs remain underutilized despite multiple initiatives. This systematic review and meta-analysis aimed to assess the pooled prevalence of intention to undergo cervical cancer screening and its associated factors among Ethiopian women, addressing a significant gap in national data.

**Methods and materials:**

This systematic review and meta-analysis followed the Preferred Reporting Items for Systematic Reviews and Meta-Analyses (PRISMA) guidelines. Databases such as PubMed, EMBASE, CINAHL, Web of Science, Cochrane Library, HINARI, Google Scholar, and African Journals online were searched using specific keywords and Medical Subject Headings (MeSH). Studies were assessed using a standardized appraisal format adapted from the Newcastle-Ottawa Scale (NOS). Data extraction and analysis were performed using Microsoft Excel-10 and STATA 17 software, respectively. Heterogeneity was evaluated with the I^2^ statistic and publication bias was examined using Egger’s test. Meta-analysis employed a random-effects model.

**Result:**

Out of the 750 articles retrieved, nine were included in this systematic review and meta-analysis. The pooled prevalence of intention to screen for cervical cancer in Ethiopia was 33% (95% CI: 9%-56%). Factors significantly associated with intention to undergo cervical cancer screening included favorable attitude (POR = 2.15, 95% CI: 1.29, 4.26), good knowledge about cervical cancer screening (POR: 3.49; 95% CI: 2.04, 6.93), and direct subjective norm (POR: 1.54; 95% CI: 1.32, 3.54).

**Conclusion:**

Based on the findings of this meta-analysis, it was observed that women’s intention toward cervical cancer screening was low. Determinants identified included favorable attitude, direct subjective norm, and good knowledge of cervical cancer screening. To enhance women’s intention for cervical cancer screening, strategies, and activities should be developed to positively influence perceptions among women and those who influence their decisions. Additionally, efforts to enhance public awareness about cervical cancer and its prevention are crucial.

## Background

Cervical cancer, a global health issue, involves the growth of malignant cells in the cervix, part of the female reproductive system [1]. Human papillomavirus (HPV) contributes significantly to its development, although it is not the sole factor. According to the International Agency for Research on Cancer Monographs, twelve types of oncogenic HPV are classified as group 1 carcinogens [2]. There are over 150 known types of HPV, with HPV 16 and 18 identified as high-risk strains responsible for half of all cases. Other risk factors include early sexual activity initiation, multiple partners, long-term oral contraceptive use, compromised immune systems, and smoking [1,3].

In 2020, cervical cancer was the fourth most commonly diagnosed cancer and the fourth leading cause of cancer-related deaths among women worldwide. Approximately 604,000 new cases and 342,000 deaths were reported [4]. It is the most frequently diagnosed cancer in 23 countries and the primary cause of cancer mortality in 36 countries, with the majority of affected nations located in sub-Saharan Africa, Melanesia, South America, and Southeast Asia [1,4].

In Africa, there were 715,000 new cancer cases and 542,000 cancer-related deaths [5]. Women in low- and middle-income countries face a 35% higher average lifetime risk of cervical cancer compared to those in high-income countries. In sub-Saharan Africa, the prevalence, death rate, and incidence rates were 27.6%, 23.2%, and 25.2%, respectively [6].

Cervical cancer represents a major health issue in Ethiopia, where it is the second most common cancer among women. Each year, there are around 7,445 new diagnoses and 5,335 deaths from the disease, according to the Information Centre on HPV and Cancer [7].

Once cervical cancer progresses to an invasive stage, its economic burden is significant. In many developing countries, cervical cancer screening is conducted only 23% of the time. Effective screening programs are crucial in reducing morbidity and mortality rates by over 80% [8]. To eliminate cervical cancer in women around the world by 2030, the World Health Organization (WHO) created a global strategy. To address these issues, 90% of women should receive the full HPV vaccine before the age of 15, 70% of women between the ages of 15 and 45 should be screened, and 90% of those who are found to have the disease should receive treatment and care [9].

Ethiopia, as a developing country, uses cost-effective methods for cervical cancer screening, such as Visual Inspection with Acetic Acid (VIA), to facilitate early detection and immediate treatment of pre-cancerous lesions. The country’s guideline recommend a ‘screen-and-treat’ strategy for women aged 30 to 49, using VIA for screening and cryotherapy for treatment. The guideline also suggests annual screenings for HIV-positive women and screenings every three years for others. However, actual screening practices are inconsistent and often rely on resource availability [10].

Despite efforts by the Ethiopian government, studies across the country revealed underutilization of cervical cancer screening programs [11–14]. Reasons mentioned include low intention to screen [15,16], poor healthcare-seeking behavior [17], poor knowledge [18], attitude toward cervical cancer [16], direct subjective norm [27], and inadequate awareness of the disease severity [17,18].

According to the latest WHO guidelines, transitioning to HPV DNA testing as the primary screening method is essential. HPV DNA tests are more effective than traditional methods like VIA or Pap smears and are less prone to human error. Adopting this approach could significantly improve cervical cancer prevention in Ethiopia, aligning with global standards and enhancing screening accuracy [19].

In conclusion, despite the critical role of enhancing screening intention for cervical cancer in improving early detection and treatment outcomes, there is a notable gap in nationally aggregated data on the prevalence and determinants of such intentions in Ethiopia [20–24]. Addressing this gap through a systematic review and meta-analysis will provide a comprehensive understanding of the factors influencing screening intention among reproductive-age women and offer valuable insights for public health strategies. This research aims to bridge the existing knowledge gap and support targeted interventions to increase screening rates and ultimately reduce cervical cancer incidence and mortality in Ethiopia.

## Methods and materials

### Registration of systematic review, data sources, and search strategies

The protocol is registered with the International Prospective Register of Systematic Reviews (PROSPERO) under registration number CRD42023440970 and can be accessed through the University of York Center for Reviews and Dissemination at https://www.crd.york.ac.uk/. This systematic review and meta-analysis was conducted in accordance with the Preferred Reporting Items for Systematic Reviews and Meta-Analyses (PRISMA) 2020 standards [25] (S1 Table).

A comprehensive search was conducted in major databases, PubMed, EMBASE, CINAHL, Google Scholar, and African Journals online, using key words and Medical Subject Headings (MeSH) specifically designed for the respective databases. Additionally, Google hand searches were carried out primarily for unpublished studies. In addition, a systematic search on Google Scholar and a manual search of institutional repositories were carried out to obtain grey literature. The Population Exposure, Comparison, and Outcomes (PECO) search algorithm was used to find the articles. For the search of the online database, the keywords ’Intention’, ’cervical cancer’, ’screening’, ’prevalence’, ’predictors’, ’women of reproductive age’, ’determinants’, ’associated factors’, and ’Ethiopia’ were used. The search terms were established, and these keywords were connected using the Boolean operators "OR" and "AND" either separately or in combination. In addition, a bibliographic search of the identified articles was carried out in order to find further publications. The most recent update of all online databases was completed on December 20, 2023. The specific search details for some major databases are provided in the S2 Table.

### Eligibility criteria

#### Inclusion criteria

The inclusion and exclusion criteria for this systematic review and meta-analysis were determined using the PICO technique.

Study design/characteristics: Observational studies (analytic cross-sectional, case-control, and cohort) that revealed the intention of screening for cervical cancer screening and its associated factors and that provided the odds ratio (OR) as a measure of the association or allowed computation of it from the data were taken into account for inclusion.

Population: Any study evaluating the prevalence of intention to screen for cervical cancer and associated factors or any of these among women of childbearing age in Ethiopia was included in the review process.

Intervention: There were no interventions or exposures investigated.

Context: Community and facility-based observational studies on the intention for cervical cancer screening and its associated factors in Ethiopia were included.

Objective/outcome: The primary outcome of this review is the pooled prevalence of women’s intention for cervical cancer screening. Secondary outcomes were predictors of the intention to screen for cervical cancer in Ethiopia.

#### Exclusion criteria

Excluded studies were those that did not provide quantitative support for the pooled estimate, as well as qualitative studies with other results of interest. Publications written in languages other than English were excluded. Moreover, qualitative studies, studies did not report outcome, and studies that do not meet the quality criteria were excluded (S3 Table).

### Data extraction

After articles were extracted from the databases, they were imported into EndNote version 9.1 for duplication management. Data extraction was carried out by two reviewers [BKL and WK] using a standardized checklist and Microsoft Excel. The details recorded included the first author’s name, publication year, study year, design, area, setup, sample size, and the percentage related to the intention to detect cervical cancer. Any disagreements encountered during the process were resolved through discussion and consensus, with input from a third reviewer [MW] to finalize decisions on article inclusion or exclusion.

### Data quality assessment

We conducted a critical evaluation of the research evidence using a standardized data evaluation format adapted from the Newcastle-Ottawa Scale (NOS) (S4 Table). This allowed us to assess the methodological quality of the study and determine the degree to which the possibility of bias in the design, conduct, and analysis of the study had been addressed. The tool has three main parts and employs a star-grading system. The validity of the research group selection procedure is taken into consideration in the first component, which can be rated up to five stars. The second part of the tool deals with the comparability of groups with potential for two stars. The last part of the grading scheme focuses on determining how each original study was exposed or what the results were. Three stars may be awarded. Following an independent evaluation of the quality of the included studies by a pair of researchers (BK and WK); any disagreements were settled by additional authors (MW). For the purposes of our research, articles with a NOS score of 5 stars out of 10 were deemed good quality [26].

### Measurement of the outcome of interest

The major outcome of this systematic review and meta-analysis was the prevalence of intention toward cervical cancer screening among reproductive age women in Ethiopia. The secondary outcome variables were predictors of the intention to screen for cervical cancer which were estimated using a pooled adjusted odds ratio (OR) with 95% confidence intervals (CI). Women were considered to have an intention for cervical cancer screening when they scored above the mean of the intention to screen for cervical cancer scale.

### Operational definitions

#### Intention

The perceived likelihood of undergoing cervical cancer screening within the next year was assessed using four items, each rated on a five-point Likert scale. Responses ranged from ’very unlikely’ (1) to ’very likely’ (5). The composite score could vary from four to twenty, with higher scores reflecting a greater intention to be screened.

#### Attitude

Individuals’ feelings and beliefs about cervical cancer screening were assessed using four items on a five-point Likert scale. The composite score ranged from four to twenty, with higher scores indicating stronger positive feelings and beliefs [16,20,21,23,24].

#### Direct subjective norm

Individuals’ views on whether they believe most people support or oppose cervical cancer screening were assessed using four items rated on a five-point Likert scale. Scores ranged from four to twenty, with higher scores indicating a stronger perceived social influence on cervical cancer screening [21,24].

#### Knowledge

It refers to the knowledge of women regarding cervical cancer in general and was assessed using 13 items. Participants’ overall knowledge was classified based on modified Bloom’s cut-off points: ’good’ if the score was between 80 and 100% (10.4–13 points), ’moderate’ if it was between 50 and 79% (6.5–10.3 points), and ’poor’ if the score was below 50% (<6.5 points) [24,27].

### Statistical methods and analysis

Forest plots and a visual evaluation of publication bias were used to represent the summary of the pooled estimates. The study characteristics of the primary studies that were considered were compiled and displayed in a table. The first author’s name, publication year, study year, study design, study area, study setup, sample size, and the percentage of intention to test for cervical cancer are all briefly shown in the summary study characteristics table. STATA^™^ version 17 was used to pool prevalence estimates and effect sizes (odds ratio) of intention for cervical cancer screening and its associated factors (S5 Table). The Egger’s [28] and Begg’s [29] tests with a p-value of less than 0.05 were used to evaluate publication bias. To estimate the pooled intention toward cervical cancer screening, a random effects model was used. The included studies were used to calculate the OR and 95% CI. We handled missing data through various strategies. We assessed the studies to identify and quantify missing information, used imputation methods when suitable, and contacted authors for clarification when possible. We also conducted sensitivity analyses to understand the impact of missing data on our findings.

### Publication bias and heterogeneity

I^2^ statistics was used to calculate the degree of heterogeneity in all studies. As a result, mild heterogeneity is defined as results of I^2^ between 0% and 40%, moderate heterogeneity between 40 and 70%, and significant heterogeneity as between 70% and 100% [30]. To evaluate publication bias, the Eggers test and funnel plot were used. It was determined that there was no publication bias due to the p-value > 0.05. Furthermore, a sensitivity analysis was performed to evaluate the potential impact of individual studies on the pooled prevalence estimates.

## Results

### Identification of the study and characteristics of included studies

**Fig 1:**
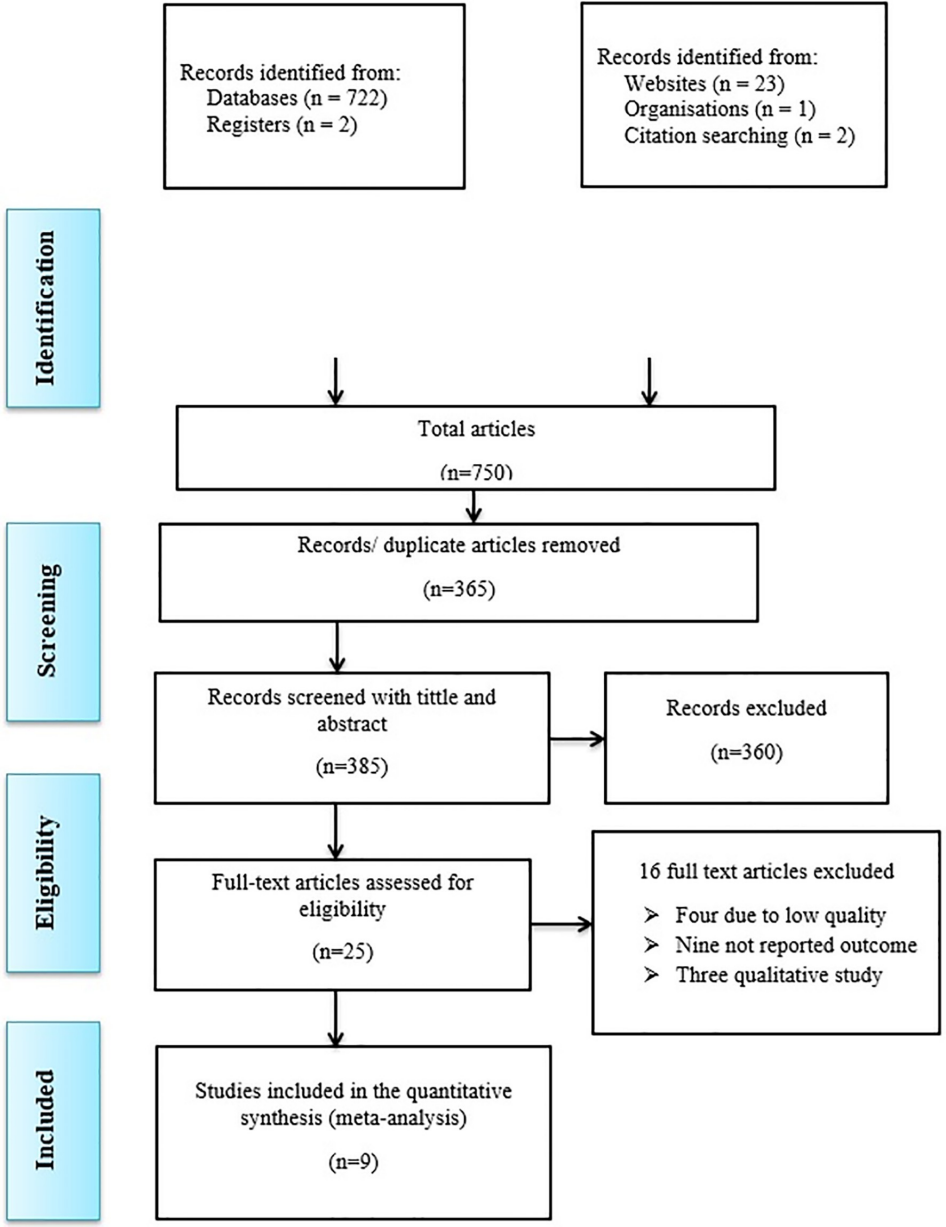
PRISMA flow chart of study selection for systematic review and meta-analysis of the intention for cervical cancer screening in Ethiopia, 2024.

Both published and unpublished studies on the intention to screen for cervical cancer in Ethiopia were included in this systematic review and meta-analysis. Out of 750 studies initially reviewed, 385 were selected based on titles and abstracts, and 365 duplicates were removed. The eligibility of 25 full-text articles was then assessed according to inclusion and exclusion criteria. Three articles were dropped due to qualitative study, four were excluded for poor quality, and nine were removed for missing the desired outcome. Thus, nine studies were included in the final quantitative meta-analysis (Fig 1).

A total of 5,362 women were included in this systematic review and meta-analysis, all from cross-sectional studies published between 2015 and 2023. The study with the largest sample size was conducted in the Girar Jarso district of Oromia, with 855 women [31], while the smallest sample was from Addis Ababa, with 333 women [32]. The studies were conducted in three regions of Ethiopia: two in Oromia, five in the Amhara region, and two in Addis Ababa [32,33]. All included studies have good quality scores on the Newcastle Ottawa Quality Assessment scale, ranging from 7 to 9 (Table 1).

**Table 1:** Characteristics of the included studies in the systematic review and meta-analysis of intention to screen for cervical cancer in Ethiopia.

S.no	Author	Period	Region	Study area	Study design	Sample size	Prevalence (%)	Study participants	Study quality
1	Wollancho W. et al.[21]	2020	Oromia	Gomma district	CBCS	422	57.3	Women aged between 15–45 years	Good
2	Eshetu, H.B. et al.[24]	2022	Amhara	Gondar	CBCS	425	38.5	Commercial sex workers	Good
3	Bishaw G. et al.[23]	2022	Amhara	East Gojjam	FBCS	424	58.3	Woman living with HIV	Good
4	Desta AA. et al.[31]	2022	Oromia	Girar Jarso district	CBCS	855	46.7	Women aged 30–65 years	Good
5	Tomas G. et al.[22]	2020	Amhara	Debre Berhan town	CBCS	821	45.3	Women aged 30–49 years	Good
6	Meried E. et al.[11]	2020	Amhara	Dabat District	CBCS	790	17.1	Women aged 18 years and above	Good
7	Alemnew A. et al[20].	2020	Amhara	Bahir Dar City	CBCS	832	55	Child Bearing Age Women	Good
8	Berhe S. et al.[33]	2023	Adiss Ababa	Adiss Ababa	FBCS	460	44.1	Female healthcare professionals	Good
9	Belete et al.[32]	2015	Adiss Ababa	Adiss Ababa	FBCS	333	62.7	HIV positive women	Good

CBCS: Community-based cross-sectional study; FBCS: Facility-based cross-sectional study.

### The prevalence of intention to screen for cervical cancer in Ethiopia

**Fig 2:**
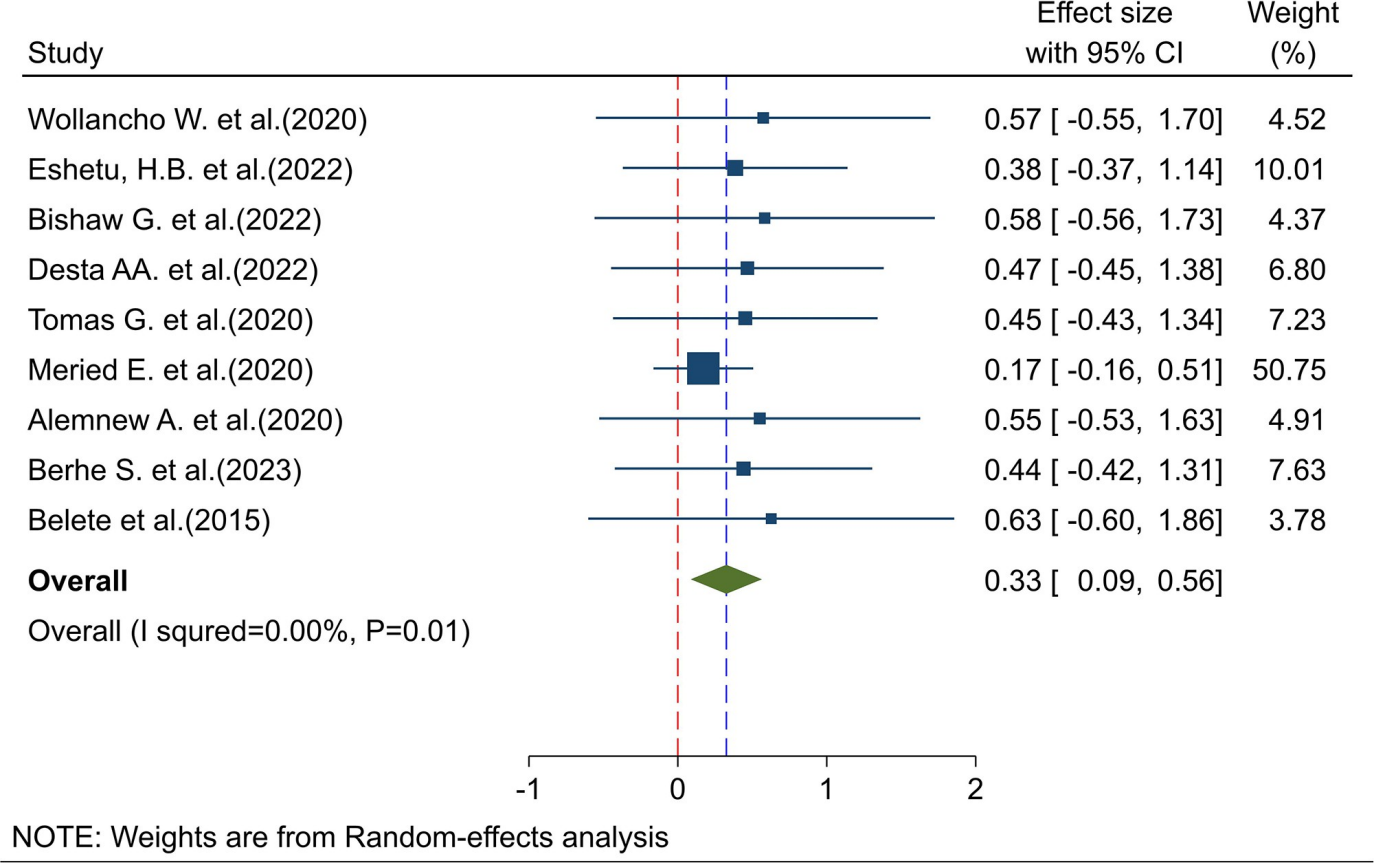
Forest plot showing the pooled prevalence of intention to screen for cervical cancer in Ethiopia, 2024.

The meta-analysis of nine studies revealed that the pooled national level of intention to screen for cervical cancer was 33% (95% CI; 9%-56%). The Cochrane heterogeneity index (I^2^ = 0.00%, P = 0.01) indicated low heterogeneity (I^2^ < 40%). These findings are illustrated in a forest plot (Fig 2).

### Publication bias

**Fig 3:**
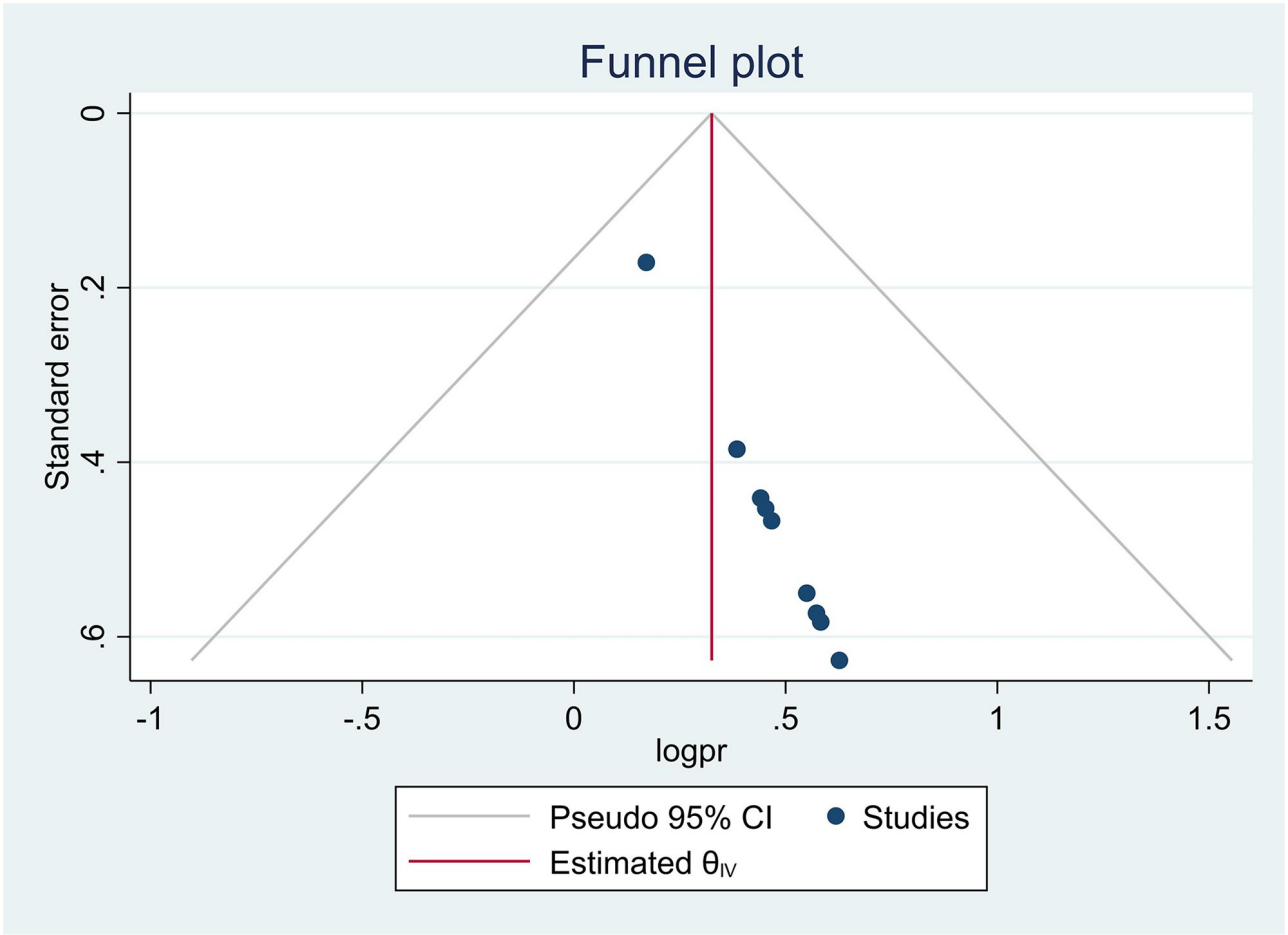
Funnel plot of the prevalence of intention to screen for cervical cancer in Ethiopia, 2024.

In this systematic review and meta-analysis, a funnel plot was used to check for publication bias at a significance level of < 0.05. Egger’s regression test P = 0.173 (p > 0.05) (statistically not significant) and the funnel plot (Fig 3) showed that there was no evidence of publication bias.

### Sensitivity analysis

**Fig 4:**
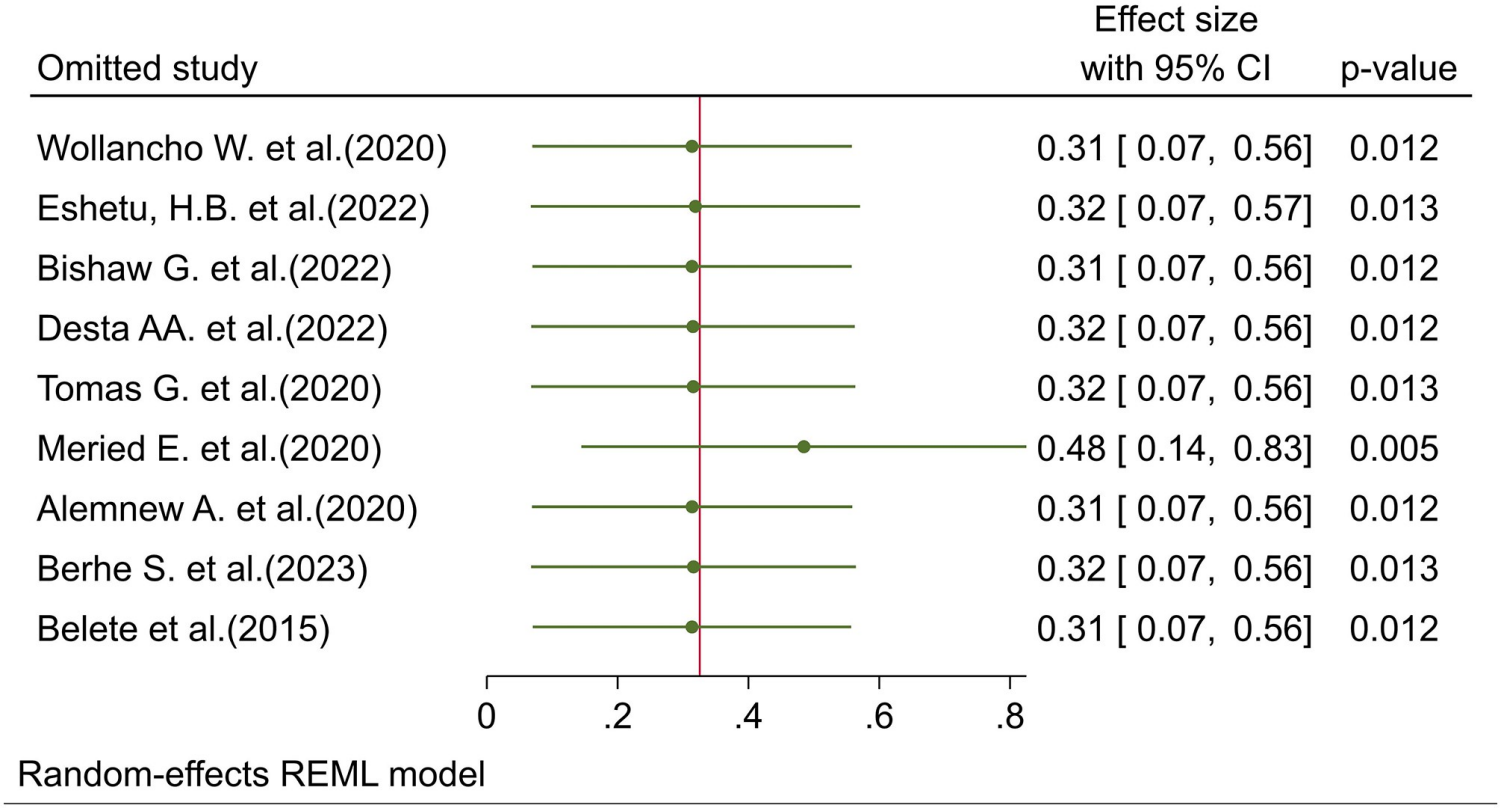
The influence graph for the meta-analysis of the results of 9 independent samples on the intention to screen for cervical cancer in Ethiopia, 2024.

A random-effects model result showed that no single study had impacted the overall pooled prevalence of intention toward cervical cancer screening (Fig 4).

### Factors associated with the intention to use cervical cancer screening in Ethiopia

#### Association of favorable attitude and intention to use cervical cancer screening

**Fig 5:**
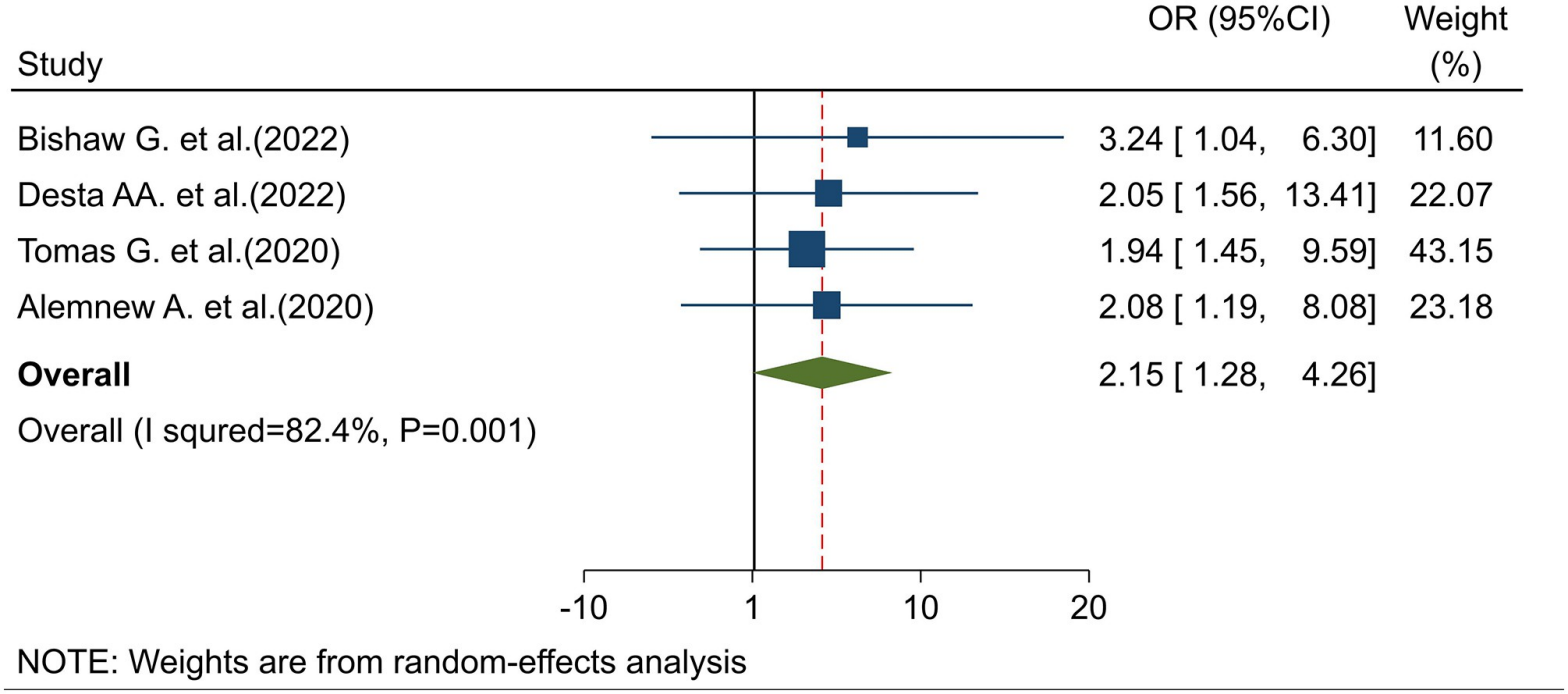
Association of attitude toward cervical cancer screening with intention to use cervical cancer screening.

The pooled effect of four studies [20,22,23,31] indicated that a favorable attitude was a key predictor of the intention to use cervical cancer screening in Ethiopia. Women with a positive attitude toward cervical cancer screening were 2.15 times more likely to intend to use screening compared to those with less favorable attitudes (POR = 2.15, 95% CI: 1.29–4.26) (Fig 5).

#### Association of knowledge with intention to use cervical cancer screening

**Fig 6:**
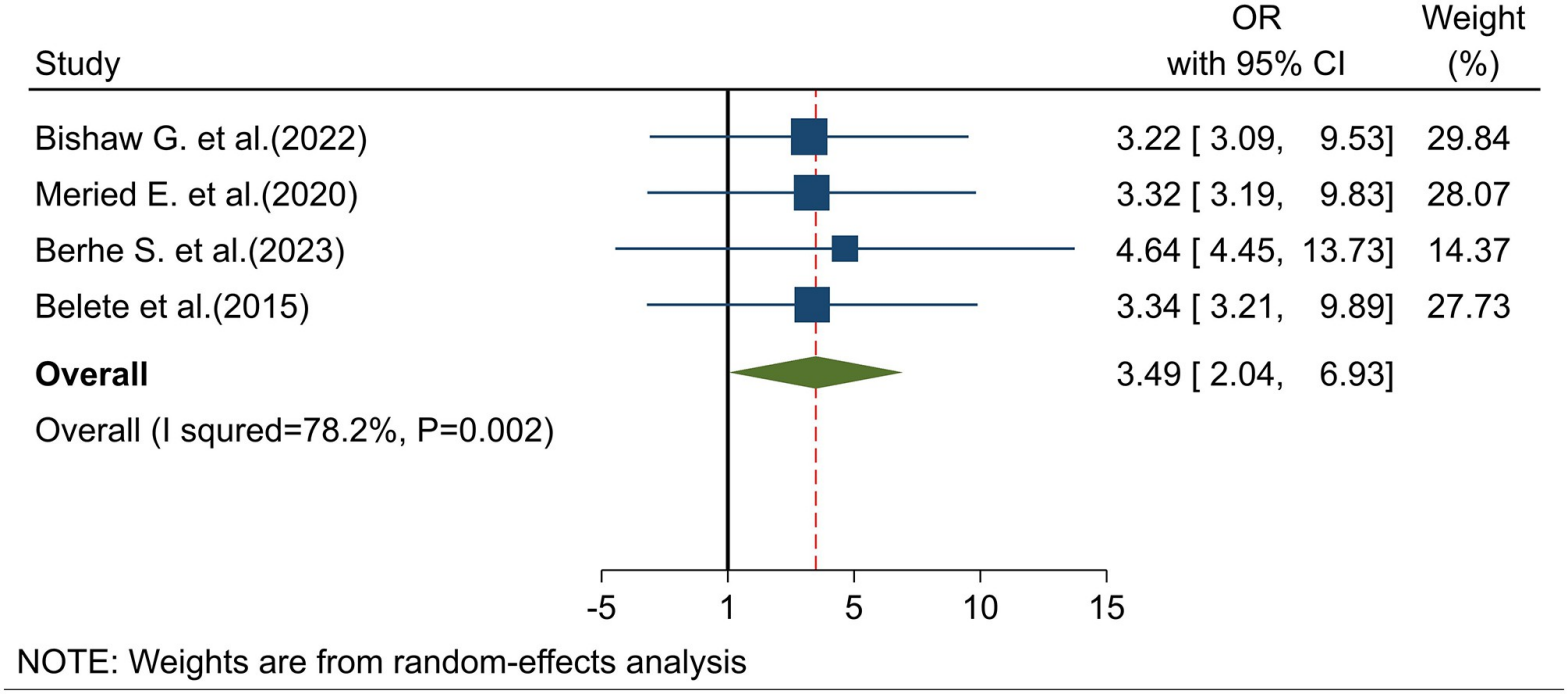
Association of knowledge about cervical cancer screening with intention to use cervical cancer screening.

Four studies [23,27,32,33] found that knowledge of cervical cancer screening was significantly associated with the intention to use screening. The pooled odds ratio revealed that women with good knowledge about cervical cancer screening were 3.5 times more likely to intend to use screening compared to those with poor knowledge (POR: 3.49; 95% CI: 2.04–6.93) (Fig 6).

#### Association of direct subjective norm with intention to use cervical cancer screening

**Fig 7:**
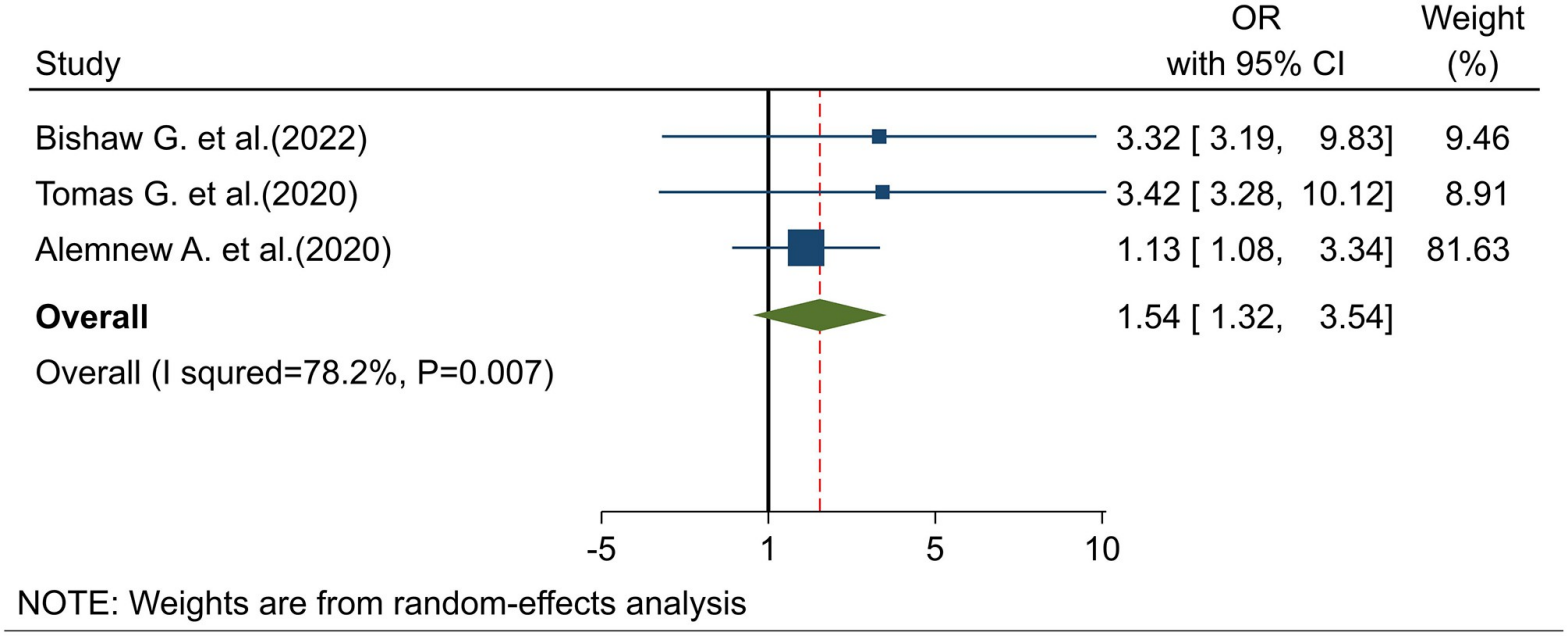
Association of direct subjective norm with intention to use cervical cancer screening.

The pooled analysis of three studies [20,22,23] showed that direct subjective norm was a key predictor of the intention to be screened. Women with a direct subjective norm were 1.54 times more likely to intend to use cervical cancer screening compared to those without such norms (POR: 1.54; 95% CI: 1.32–3.54) (Fig 7).

## Discussion

Based on the available information, this systematic review and meta-analysis is the first of its kind conducted at the national level to evaluate the determinants of cervical cancer screening intentions among women of reproductive age in Ethiopia. According to this systematic review and meta-analysis, women’s intention to undergo cervical cancer screening was found to be low. The key determinants identified were a favorable attitude, direct subjective norms, and strong knowledge about cervical cancer screening.

This systematic review and meta-analysis found that the pooled prevalence of cervical cancer screening intention among women in Ethiopia is 33% (95% CI: 9%-56%). This rate is closer to Japan’s 29% [34] but lower than the higher rates observed in China (53.8%) [35], Uganda (63%) [36], Ghana (82%) [37], and the United Kingdom (74%) [38].

The higher rates in China, Uganda, Ghana, and the UK reflect more effective public health initiatives and better healthcare systems. In contrast, Ethiopia’s lower rate may be due to similar constraints faced in Japan [34], such as limited healthcare access and public awareness. These discrepancies might also stem from methodological issues, including the small number of studies and limitations in publication bias assessment, which could skew estimates [39]. Future research with a larger sample and improved methods is needed for more accurate results.

In the current review, HIV-positive women showed a higher level of intention to screen compared to other women. This might be attributed to the Ethiopian guidelines’ focus on annual screenings for HIV-positive individuals, reflecting their increased susceptibility to cervical cancer and potentially driving greater awareness and screening participation [32].

This review highlights that a positive attitude toward cervical cancer screening is a significant predictor of screening intention, consistent with findings from Iran [40], where intention was identified as the strongest predictor of screening behavior. Women who perceive cervical cancer screening as an effective tool for early detection and treatment are more likely to develop both a positive attitude and a strong intention to participate in screening programs. This aligns with the broader understanding that positive beliefs about screening can drive higher uptake [41]. However, this finding contrast with studies conducted in Latin America [42] and Canada [43], where differing results may be due to variations in public awareness, informational resources, or cultural contexts. These discrepancies underscore the need for targeted efforts to improve women’s attitudes toward cervical cancer screening globally, as fostering a positive outlook is crucial for enhancing screening intentions and ultimately increasing screening rates.

Our analysis also revealed that understanding cervical cancer screening significantly influences the intention to get screened. Women with better knowledge were more likely to intend to participate in screening compared to those with less knowledge. This finding is consistent with research from Eastern Uganda [44], Nigeria [45], and Tanzania [46], which indicates that increased awareness about cervical cancer and its prevention drives higher screening intentions. This is scientifically supported by the fact that informed individuals grasp the benefits of early detection, the preventive role of screening, and the importance of timely treatment, which collectively encourage proactive health decisions.

The direct subjective norm was also a significant predictor of the intention to undergo screening. Women with a strong direct subjective norm were more likely to intend to use cervical cancer screening compared to their counterparts. This finding aligns with research from Singapore and Iran, where subjective norms primarily predict screening intentions [47,48] as well as a study on low-income women [49]. This suggests that community members, traditional birth attendants, family, friends, husbands, and healthcare professionals play a crucial role in raising awareness and encouraging cervical cancer screening. However, this result contrasts with a Canadian study that found subjective norms do not predict screening intentions [50]. The observed discrepancies may be due to sociocultural differences between the study populations and those in developed nations.

The reason for the strengths of our study is that the search strategy and data abstraction were carried out according to a predetermined methodology and the quality of each individual research piece was assessed using widely recognized instruments for a critical appraisal system. Comprehensive systematic search was utilized for this systematic review and meta-analysis, which included studies without specifying the publication year or demographic data.

However, this systematic review and meta-analysis has some limitations. The representativeness of the findings may be compromised due to the review’s focus on studies from only three regions, including two of the nine regional states and the capital city. Additionally, the small number of studies included and the small sample sizes in some of the research could affect the reliability of the results and the precision of the estimates. These factors may limit the generalizability of the findings and the overall impact of the review.

## Conclusion

The meta-analysis indicated that women’s intention to undergo cervical cancer screening in Ethiopia was low, influenced by factors such as attitude, subjective norms, and knowledge. To boost screening intentions and reduce morbidity and mortality, it is essential to implement targeted educational programs to enhance knowledge about cervical cancer and its screening benefits. Additionally, fostering positive attitudes through specific interventions and strengthening community-based initiatives to improve social support and norms are crucial. Policies should focus on increasing accessibility by reducing financial and logistical barriers and establishing monitoring systems to evaluate program effectiveness. Raising awareness about the availability of screening services will further help maximize participation rates and reduce the disease burden. Future research should utilize longitudinal data to track how changes in predictors affect screening intentions and behaviors, and to evaluate the effectiveness of various interventions in increasing screening uptake.

## Supporting information

S1 Table PRISMA 2020 main checklist. (DOCX)

S2 Table Search strings used for a comprehensive search in major databases. (DOCX)

S3 Table Reasons for the excluded primary studies from the review. (DOCX)

S4 Table NOS-checklist for systematic reviews and research syntheses. (DOCX)

S5 Table Data set. (XLSX)

## Abbreviations

CI: Confidence intervals
HPV: Human Papilloma, various
POR: Pooled Odds Ratio
PRISMA: Preferred Reporting Items for Systematic Reviews and Meta-Analyses
