# Supplementary material for: Determinants of cervical cancer screening intention among reproductive age women in Ethiopia: A systematic review and meta-analysis
Source: PLoS One. 2024 Oct 31;19(10):e0312449. doi: 10.1371/journal.pone.0312449 (PMC11527304; doi:10.1371/journal.pone.0312449)
Supplement: S2 Table — (DOCX) [file pone.0312449.s002.docx]

| S2 Table: Search strings used for a comprehensive search in databases |
| --- |
| PubMed: (("Intention to Screen"[Mesh] OR "Intention to Use"[Mesh] OR "Intention to Undergo"[Mesh] OR "Intention to Participate"[Mesh]) AND ("Uterine Cervical Neoplasms/diagnosis"[Mesh] OR "Papanicolaou Test"[Mesh] OR "Mass Screening"[Mesh]) AND (Ethiopia[Mesh] OR Ethiopia*) AND ("Reproductive Age"[Mesh] OR "Adolescent"[Mesh])) AND (("Women"[Mesh] OR "Female"[Mesh])) |
|  |
| Ovid (MEDLINE): (("Intention to Screen".mp. OR "Intention to Use".mp. OR "Intention to Undergo".mp. OR "Intention to Participate".mp.) AND ("Uterine Cervical Neoplasms/diagnosis" OR "Papanicolaou Test" OR "Mass Screening") AND (Ethiopia OR Ethiopia*) AND ("Reproductive Age" OR "Childbearing Age" OR "Fertile Age") AND (Women OR Female*)) |
|  |
| EMBASE: ('cervical cancer screening'/exp OR 'pap smear'/exp OR 'pap test'/exp) AND ('intention'/exp OR 'intention to use'/exp OR 'intention to undergo'/exp OR 'intention to participate'/exp) AND ('reproductive age'/exp OR 'childbearing age'/exp OR 'fertile age'/exp) AND ('women'/exp OR 'female'/exp) AND ('Ethiopia'/exp) |
|  |
| HINARI: ((SubjectTerms:(cervical cancer screening)) OR (Pap smear) OR (Pap test)) AND ((SubjectTerms:(intention)) OR (intention to use) OR (intention to undergo) OR (intention to participate)) AND ((SubjectTerms:(reproductive age)) OR (childbearing age) OR (fertile age)) AND ((SubjectTerms:(women)) OR (female)) AND ((SubjectTerms:(Ethiopia))) |
|  |
| Google Scholar: ("intention to use" OR "intention to undergo" OR "intention to participate") AND ("cervical cancer screening" OR "Pap smear" OR "Pap test") AND (Ethiopia OR Ethiopian) AND ("reproductive age" OR "childbearing age" OR "fertile age") AND (women OR females) |
