# Supplementary material for: Determinants of cervical cancer screening intention among reproductive age women in Ethiopia: A systematic review and meta-analysis
Source: PLoS One. 2024 Oct 31;19(10):e0312449. doi: 10.1371/journal.pone.0312449 (PMC11527304; doi:10.1371/journal.pone.0312449)
Supplement: S3 Table — (DOCX) [file pone.0312449.s003.docx]

S3 Table: A table of all studies identified in the literature search

| No | Author | Title | Reference URL | Main reason for exclusion |
| --- | --- | --- | --- | --- |
|  | Belay, A.S., et al. | Cervical cancer screening utilization and its predictors among women in bench Sheko Zone, Southwest Ethiopia: using health belief model | https://doi.org/10.1186/s12885-023-10927-x | Outcome of interest not available |
|  | Wollancho W. et al. | Determining behavioral intention and its predictors towards cervical cancer screening among women in Gomma district, Jimma, Ethiopia: Application of the theory of planned behavior | https://doi.org/10.1371/journal. pone.0238472 | Included |
|  | Amado G, et al. | Cervical cancer screening practices and its associated factors among females of reproductive age in Durame town, Southern Ethiopia | http:// doi: 10.1371/journal.pone.0279870 | Outcome of interest not available |
|  | Belete et al. | Willingness and acceptability of cervical cancer screening among women living with HIV/AIDS in Addis Ababa, Ethiopia: a cross sectional study | DOI 10.1186/s40661-015-0012-3 | Included |
|  | Demissie BW, et al. | Communities’ perceptions towards cervical cancer and its screening in Wolaita zone, southern Ethiopia: A qualitative study | https://doi.org/10.1371/journal.pone.0262142 | Qualitative study |
|  | Gebeyehu et al. | Knowledge, Awareness, and Willingness of women living with HIV on follow-up at Jinka General Hospital Regarding Cervical Cancer Screening: The Application of a Health Belief Model | http://dx.doi.org/10.1186/ s12905-015-0243-9 | Outcome of interest not available |
|  | Eshetu, H.B. et al. | Predictors of intention to receive cervical cancer screening among commercial sex workers in Gondar city, northwest Ethiopia: application of the theory of planned behavior | https://doi.org/10.1186/s12905-022-02055-8 | Included |
|  | Bishaw G. et al. | Intention to Use Cervical Cancer Screening Service Among Womans Living With HIV Who Attends to ART Clinic, Amahara East Gojjam, Ethiopia 2022 | DOI: 10.35248/2167-  0420.23.12.630 | Included |
|  | Tenna et al. | Predictors of intention to use cervical cancer screening, among women’s attending maternal and child health service in Yirgalem town health institutions SNNPR Ethiopia, the theory of planned behavior perspective | [http://10.140.5.162//handle/123456789/729](http://10.140.5.162/handle/123456789/729) | Poor quality |
|  | Kahasay et al. | The assessment of knowledge, attitude and intention to use cervical cancer screening and its correlates among army women in Ethiopia | <http://etd.aau.edu.et/handle/123456789/6847> | Poor quality |
|  | Abamecha et al. | Psychographic predictors of intention to use cervical cancer screening services among women attending maternal and child health services in Southern Ethiopia: the theory of planned behavior (TPB) perspective | https://doi.org/10.1186/s12889-019-6745-x | Poor quality |
|  | Megersa et al. | Community cervical cancer screening: Barriers to successful home-based HPV self-sampling in Dabat district, North Gondar, Ethiopia. A qualitative study | https://doi.org/10.1371/journal.pone.0243036 | Outcome of interest not available (Qualitative study) |
|  | Gebru Z., et al. | Barriers to Cervical Cancer Screening in Arba Minch Town, Southern Ethiopia: A Qualitative Study | [http:// doi:10.4172/2161-0711.1000401](http://10.0.3.248/j.jadohealth.2020.02.023) | Outcome of interest not available (Qualitative study) |
|  | Alemnew A. et al | Intention to Screen for Cervical Cancer Among Child Bearing Age Women in Bahir Dar City, NorthWest Ethiopia: Using Theory of Planned Behaviour | https://doi.org/10.2147/IJWH.S277441 | Included |
|  | Ruddies, F., et al. | Cervical cancer screening in rural Ethiopia: a cross- sectional knowledge, attitude and practice study | - https://doi.org/10.1186/s12885-020-07060-4 | Outcome of interest not available |
|  | Burrowes, S., et al. | Perceptions of cervical cancer care among Ethiopian women and their providers: a qualitative study | https://doi.org/10.1186/s12978-021-01316-3 | Outcome of interest not available (Qualitative study) |
|  | Ayanto, S. Y., et al. | Women’s and health professionals’ perceptions, beliefs and barriers to cervical cancer screening uptake in Southern Ethiopia: a qualitative study | https://doi.org/10.1080/26410397.2023.2258477 | Qualitative study |
|  | Habtu, Y., et al. | Health seeking behavior and its determinants for cervical cancer among women of childbearing age in Hossana Town, Hadiya zone, Southern Ethiopia: community based cross sectional study | https://doi.org/10.1186/s12885-018-4203-2 | Outcome of interest not available |
|  | Birhanu Z., et al. | Health seeking behavior for cervical cancer in Ethiopia: a qualitative study. | https://doi.org/10.1186/1475-9276-11-83 | Qualitative study |
|  | Desta AA. et al. | Willingness to utilize cervical cancer screening among Ethiopian women aged 30–65 years | https://doi.org/10.3389/fgwh.2022.939639 | Included |
|  | Tomas G. et al. | Intention to Screen for Cervical Cancer in Debre Berhan Town, Amhara Regional State, Ethiopia: Application of Theory of Planned Behavior | https://doi.org/10.1155/2020/3024578 | Included |
|  | Aweke YH, et al. | Knowledge, attitude and practice for cervical cancer prevention and control among women of childbearing age in Hossana Town, Hadiya zone, Southern Ethiopia: Community-based cross-sectional study | https://doi.org/10.1371/journal.pone.0181415 | Outcome of interest not available |
|  | Meried E. et al. | Cervical Cancer Screening Acceptance among Women in Dabat District, Northwest Ethiopia, 2017: An Institution-Based Cross-Sectional Study | https://doi.org/10.1155/2020/2805936 | Included |
|  | Berhe S. et al. | Intention to screen for cervical cancer and factors associated among female healthcare professionals using the trans-theoretical model: Addis Ababa, Ethiopia | https://www.ajol.info/index.php/ejhd/article/view/255734 | Included |
|  | Ketema B., et al. | Does Prior Experience Matter? Intention to Undergo Cervical Cancer Screening among Rural Women in South-Central Ethiopia | https://doi.org/10.3390/curroncol31090363 | Poor quality |
|  | Ayanto et al. | Determinants of cervical cancer screening utilization among women in Southern Ethiopia | https://doi: 10.1038/s41598-022-18978-z. | Outcome of interest not available |
|  | Derbie et al. | Cervical cancer in Ethiopia: a review of the literature | https://doi: 10.1007/s10552-022-01638-y | Outcome of interest not available |
|  | Derbie et al. | Human papillomavirus in Ethiopia | doi: 10.1007/s13337-019-00527-4 | Outcome of interest not available |
|  | Wakwoya et al. | Precancerous cervical lesion screening acceptance among women in Eastern Ethiopia | doi: 10.1136/bmjopen-2023-073721 | Outcome of interest not available |
|  | Yimer et al. | Cervical cancer screening uptake in Sub-Saharan Africa: a systematic review and meta-analysis | doi: 10.1016/j.puhe.2021.04.014. | Outcome of interest not available |
|  | Getachew et al. | Cervical cancer screening knowledge and barriers among women in Addis Ababa, Ethiopia | doi: 10.1371/journal.pone.0216522. eCollection 2019. | Outcome of interest not available |
|  | Ruddies et al. | Cervical cancer screening in rural Ethiopia: a cross- sectional knowledge, attitude and practice study | doi: 10.1186/s12885-020-07060-4. | Outcome of interest not available |
|  | Desta et al. | Cervical cancer screening utilization and predictors among eligible women in Ethiopia: A systematic review and meta-analysis | doi: 10.1371/journal.pone.0259339. | Outcome of interest not available |
|  | Lim et al. | Barriers to utilisation of cervical cancer screening in Sub Sahara Africa: a systematic review | doi: 10.1111/ecc.12444. Epub 2016 Feb 7. | Review |
|  | McCormick et al. | Cervical Cancer Screening Positivity Among Women Living With HIV in CDC-PEPFAR Programs 2018-2022 | doi: 10.1097/QAI.0000000000003286. | Outcome of interest not available |
|  | Belay et al. | Cervical cancer screening utilization and its predictors among women in bench Sheko Zone, Southwest Ethiopia: using health belief model | doi: 10.1186/s12885-023-10927-x. | Outcome of interest not available |
|  | Mesfin et al. | Usage of cervical cancer screening services among HIV-positive women in Southern Ethiopia: a multicentre cross-sectional study | doi: 10.1136/bmjopen-2022-068253. | Outcome of interest not available |
|  | Assefa et al. | Determinants of cervical cancer screening utilization among women attending health facilities of Dessie town, Northeast Ethiopia | doi: 10.1186/s12885-022-10447-0. | Outcome of interest not available |
|  | Endalamaw et al. | Improving cervical cancer continuum of care towards elimination in Ethiopia: a scoping review | doi: 10.1007/s10552-023-01813-9. | Outcome of interest not available |
|  | Demissie et al. | Communities' perceptions towards cervical cancer and its screening in Wolaita zone, southern Ethiopia: A qualitative study | doi: 10.1371/journal.pone.0262142. eCollection 2022 | Qualitative |
|  | Jemal et al. | Cervical cancer screening utilization and associated factors among female health workers in public health facilities of Hossana town, southern Ethiopia: A mixed method approach | doi: 10.1371/journal.pone.0286262. eCollection 2023. | Qualitative |
|  | Natae et al. | Cervical cancer screening uptake and determinant factors among women in Ambo town, Western Oromia, Ethiopia: Community-based cross-sectional study | doi: 10.1002/cam4.4369. Epub 2021 Oct 27. | Outcome of interest not available |
|  | Tesfaye et al. | Cervical cancer screening uptake and associated factors among Women Living with Human Immunodeficiency Virus in public hospitals, eastern Ethiopia | doi: 10.3389/fonc.2023.1249151. eCollection 2023. | Outcome of interest not available |
|  | Zewdie et al | Determinants of late-stage cervical cancer presentation in Ethiopia: a systematic review and meta-analysis | doi: 10.1186/s12885-023-11728-y. | Review |
|  | Ashenafi et al. | Characteristics of Women Seeking Cervical Cancer Cytology Screening in a Private Health Facility | doi: 10.3390/medicina59091624. | Outcome of interest not available |
|  | Gizaw et al. | Uptake of cervical cancer screening and its predictors among women of reproductive age in Gomma district, South West Ethiopia: a community-based cross-sectional study | doi: 10.1186/s13027-022-00455-x. | Outcome of interest not available |
|  | Kassa et al. | Survival of women with cervical cancer in East Africa: a systematic review and meta-analysis | doi: 10.1080/01443615.2023.225330. | Review article |
|  | Gizaw et al. | Uptake of Cervical Cancer Screening in Ethiopia by Self-Sampling HPV DNA Compared to Visual Inspection with Acetic Acid: A Cluster Randomized Trial | doi: 10.1158/1940-6207.CAPR-19-0156. | Outcome of interest not available |
|  | Mengesha et al. | Uptake and barriers to cervical cancer screening among human immunodeficiency virus-positive women in Sub Saharan Africa: a systematic review and meta-analysis | doi: 10.1186/s12905-023-02479-w. | Outcome of interest not available |
|  | Legasu et al. | Determinants of cervical cancer screening service utilization among women attending healthcare services in Amhara region referral hospitals: a case-control study | doi: 10.1186/s12905-022-02071-8 | Outcome of interest not available |
|  | Tesfaye et al. | Cervical cancer screening practice and associated factors among women employees in Wolaita Zone hospitals, Southern Ethiopia, 2017: cross-sectional study | doi: 10.11604/pamj.2022.42.318.34675 | Outcome of interest not available |
|  | Shero et al. | Cervical cancer screening utilization and associated factors among women attending antenatal care at Asella Referral and Teaching Hospital, Arsi zone, South Central Ethiopia | doi: 10.1186/s12905-023-02326-y. | Outcome of interest not available |
|  | Endale et al. | MiRNA in cervical cancer: Diagnosis to therapy: Systematic review | doi: 10.1016/j.heliyon.2024.e24398. | Review, Outcome of interest not available |
|  | Amado et al. | Cervical cancer screening practices and its associated factors among females of reproductive age in Durame town, Southern Ethiopia | doi: 10.1371/journal.pone.0279870. | Outcome of interest not available |
|  | Biazin et al. | Concordance of Anyplex™ II HPV HR assays with reference HPV assays in cervical cancer screening: Systematic review | doi: 10.1016/j.jviromet.2021 | Outcome of interest not available, review |
|  | Mohamed et al. | Cervical Cancer Screening Service Utilization and Associated Factors Among Women Living With HIV Receiving Anti-Retroviral Therapy at Adama Hospital Medical College, Ethiopia | doi: 10.1177/23779608231152072. | Outcome of interest not available |
|  | Tsegaye et al. | Evaluation of cervical cancer screening program in Gondar city administration public health facilities, Northwest Ethiopia, 2021: mixed method approach | doi: 10.1186/s12885-023-11533-7. | Outcome of interest not available |
|  | Gizaw et al. | Reasons for Not Attending Cervical Cancer Screening and Associated Factors in Rural Ethiopia | doi: 10.1158/1940-6207.CAPR-19-0485. | Outcome of interest not available |
|  | Gebremeskel et al. | Determinants of cervical cancer screening utilization among HIV-positive women, in public general hospitals of Central Zone, Tigray, Ethiopia, 2020: Case-control study | doi: 10.1371/journal.pone.0289042. | Outcome of interest not available |
|  | Midaksa et al. | Women's sexual autonomy as a determinant of cervical cancer screening uptake in Addis Ababa, Ethiopia: a case-control study | doi: 10.1186/s12905-022-01829-4. | Outcome of interest not available |
|  | Deressa et al. | Contemporary treatment patterns and survival of cervical cancer patients in Ethiopia | doi: 10.1186/s12885-021-08817-1 | Outcome of interest not available |
|  | Gebisa et al. | Knowledge, Attitude, and Practice Toward Cervical Cancer Screening Among Women Attending Health Facilities in Central Ethiopia | doi: 10.1177/10732748221076680 | Outcome of interest not available |
|  | Dirirsa et al. | Utilization of cervical cancer screening service and associated factors among women of reproductive age group in Ilu Abba Bor zone, southwestern Ethiopia, 2019: Cross-sectional study | doi: 10.1177/20503121221094904. | Outcome of interest not available |
|  | Lemma et al. | Determinants of utilization of cervical cancer screening among women in the age group of 30-49 years in Ambo Town, Central Ethiopia: A case-control study | doi: 10.1371/journal.pone.0270821. | Outcome of interest not available |
|  | Gebrekiristos et al. | Determinants of Cervical Cancer Screening Service Utilization Among HIV-Positive Women Aged 25 Years and Above Attending Adult ART Clinics in Southern Tigray, Ethiopia | doi: 10.1177/10732748221126944. | Outcome of interest not available |
|  | Jibat et al. | Less known but greatly feared: Cervical cancer in Ethiopia community awareness | doi: 10.1016/j.heliyon.2024.e28328. | Outcome of interest not available |
|  | Lulu et al. | Factors affecting cervical cancer screening service uptake among clients visiting the ART clinic at Hawassa University Comprehensive Specialized Hospital, Hawassa, Ethiopia: A cross-sectional study | doi: 10.1016/j.jcpo.2023.100422. | Outcome of interest not available |
|  | Tefera et al. | Uptake of Cervical Cancer Screening and Associated Factors Among 15-49-Year-Old Women in Dessie Town, Northeast Ethiopia | doi: 10.1007/s13187-016-1021-6. | Outcome of interest not available |
|  | Saleem et al. | Knowledge and awareness of cervical cancer in Southwestern Ethiopia is lacking: A descriptive analysis | doi: 10.1371/journal.pone.0215117. | Outcome of interest not available |
|  | Ayanto et al. | Effectiveness of couple education and counseling on knowledge, attitude and uptake of cervical cancer screening service among women of child bearing age in Southern Ethiopia: A cluster randomized trial protocol | doi: 10.1371/journal.pone.0270663. | Protocol |
|  | Emru et al. | Role of awareness on cervical cancer screening uptake among HIV positive women in Addis Ababa, Ethiopia: A cross-sectional study | doi: 10.1177/17455065211017041. | Outcome of interest not available |
|  | Destaw et al. | Cervical cancer screening "see and treat approach": real-life uptake after invitation and associated factors at health facilities in Gondar, Northwest Ethiopia | doi: 10.1186/s12885-021-08761-0. | Outcome of interest not available |
|  | Chipanta et al. | Socioeconomic inequalities in cervical precancer screening among women in Ethiopia, Malawi, Rwanda, Tanzania, Zambia and Zimbabwe: analysis of Population-Based HIV Impact Assessment surveys | doi: 10.1136/bmjopen-2022-067948. | Different outcome |
|  | Belay et al. | Cervical Cancer Screening Utilization and Associated Factors Among Women Aged 30 to 49 Years in Dire Dawa, Eastern Ethiopia | doi: 10.1177/1073274820958701. | Different outcome |
|  | Ayanto et al. | Women's and health professionals' perceptions, beliefs and barriers to cervical cancer screening uptake in Southern Ethiopia: a qualitative study | doi: 10.1080/26410397.2023.2258477. | Qualitative study |
|  | Kasim et al. | Cervical Cancer Screening Service Utilization and Associated Factors among Women in the Shabadino District, Southern Ethiopia | doi: 10.1155/2020/6398394. | Outcome of interest not available |
|  | Eshetu et al. | Predictors of intention to receive cervical cancer screening among commercial sex workers in Gondar city, northwest Ethiopia: application of the theory of planned behavior | doi: 10.1186/s12905-022-02055-8. | Included |
|  | Yirsaw et al. | Cervical cancer screening utilization and associated factors among women living with HIV in Ethiopia, 2024: systematic review and meta-analysis | doi: 10.1186/s12905-024-03362-y. | Outcome of interest not available |
|  | Muluneh et al. | Predictors of cervical cancer screening service utilization among commercial sex workers in Northwest Ethiopia: a case-control study | doi: 10.1186/s12905-019-0862-7. | Outcome of interest not available |
|  | Feyisa et al. | Cervical Cancer Progression in Patients Waiting for Radiotherapy Treatment at a Referral Center in Ethiopia: A Longitudinal Study | doi: 10.1200/GO.22.00435. | Different outcome |
|  | Hambisa et al. | Common predictors of cervical cancer related mortality in Ethiopia. A systematic review and meta-analysis | doi: 10.1186/s12889-024-18238-x. | Review |
|  | Gebremariam et al. | Breast and cervical cancer patients' experience in Addis Ababa city, Ethiopia: a follow-up study protocol | doi: 10.1136/bmjopen-2018-027034. | Protocol |
|  | Nega et al. | Low uptake of cervical cancer screening among HIV positive women in Gondar University referral hospital, Northwest Ethiopia: cross-sectional study design | doi: 10.1186/s12905-018-0579-z. | Different outcome |
|  | Mekonnen et al. | Cervical Cancer Screening Uptake and Associated Factors among HIV-Positive Women in Ethiopia: A Systematic Review and Meta-Analysis | doi: 10.1155/2020/7071925. | Review |
|  | Getaneh et al. | Knowledge, attitude and practices on cervical cancer screening among undergraduate female students in University of Gondar, Northwest Ethiopia: an institution based cross sectional study | doi: 10.1186/s12889-021-10853-2. | Outcome of interest not available |
|  | Bayu et al. | Cervical Cancer Screening Service Uptake and Associated Factors among Age Eligible Women in Mekelle Zone, Northern Ethiopia, 2015: A Community Based Study Using Health Belief Model | doi: 10.1371/journal.pone.0149908. | Outcome of interest not available |
|  | Sharama et al. | No woman left behind: achieving cervical cancer elimination among women living with HIV | doi: 10.1016/S2352 3018(23)00082-6. | Outcome of interest not available |
|  | Megersa et al. | Community cervical cancer screening: Barriers to successful home-based HPV self-sampling in Dabat district, North Gondar, Ethiopia. A qualitative study | doi: 10.1371/journal.pone.0243036. | Qualitative |
|  | Alie et al. | Determinants of Cervical Cancer Screening Among Women Aged 30-49 Years Old in Four African Countries: A Cross-Sectional Secondary Data Analysis | doi: 10.1177/10732748231195681. | Outcome of interest not available |
|  | Taddese et al. | Cervical Cancer Screening Practice Among Women: A Community Based Cross-Sectional Study Design | doi: 10.1177/00469580231159743. | Outcome of interest not available |
|  | Nigussie et al. | Cervical cancer screening service utilization and associated factors among age-eligible women in Jimma town using health belief model, South West Ethiopia | doi: 10.1186/s12905-019-0826-y. | Outcome of interest not available |
|  | Tesfaw et al. | Utilisation of cervical cancer screening and factors associated with screening utilisation among women aged 30-49 years in Mertule Mariam Town, East Gojjam Zone, Ethiopia, in 2021: a cross-sectional survey | doi: 10.1136/bmjopen-2022-067229. | Outcome of interest not available |
|  | Gelassa et al. | Knowledge and practice of cervical cancer screening and its associated factors among women attending maternal health services at public health institutions in Assosa Zone, Benishangul-Gumuz, Northwest Ethiopia, 2022: a cross-sectional study | doi: 10.1136/bmjopen-2022-068860. | Outcome of interest not available |
|  | Bekelle et al. | Knowledge, Attitude, and Practice Toward Cervical Cancer Screening and Associated Factors Among College and University Female Students in Dire Dawa City, Eastern Ethiopia | doi: 10.1177/11769351221084808. | Outcome of interest not available |
|  | Ziyad et al. | Determinants of Cervical Cancer Screening among Female Health Professionals in Harar Town, Eastern Ethiopia: A Cross-Sectional Study | doi: 10.1155/2024/1430978. | Outcome of interest not available |
|  | Beyene et al. | Male support for cervical cancer screening in Debre Berhan City Ethiopia a community based cross sectional survey | doi: 10.1038/s41598-024-69439-8 | Outcome of interest not available |
|  | Lott et al. | Health workers' perspectives on barriers and facilitators to implementing a new national cervical cancer screening program in Ethiopia | doi: 10.1186/s12905-021-01331-3 | Outcome of interest not available |
|  | Burt et al. | Cervix Cancer in Sub-Saharan Africa: An Assessment of Cervical Cancer Management | doi: 10.1200/GO.20.00079 | Outcome of interest not available |
|  | Teka et al. | A Technical Comparison of Human Papillomavirus Genotyping Assays from a Population-Based Cervical Cancer Screening in South Central Ethiopia | doi: 10.2147/CMAR.S360712 | Outcome of interest not available |
|  | Bogale et al. | Performance of visual Inspection With Acetic Acid for Cervical Cancer Screening as Compared to Human papillomavirus Deoxyribonucleic acid Testing Among Women With HIV in Ethiopia: A Comparative Cross-sectional Study | doi: 10.1177/10732748221114980 | Outcome of interest not available |
|  | Gabaa et al. | Utilization and outcomes of cervical cancer screening services in Harare City, 2012-2016: a secondary data analysis | doi: 10.1186/s12913-019-4314-8 | Outcome of interest not available |
|  | Ayenew et al. | Uptake of cervical cancer screening service and associated factors among age-eligible women in Ethiopia: systematic review and meta-analysis | doi: 10.1186/s13027-020-00334-3 | Review article |
|  | Desta et al. | Willingness to utilize cervical cancer screening among Ethiopian women aged 30-65 years | doi: 10.3389/fgwh.2022.939639 | Outcome of interest not available |
|  | Ararsa et al. | Knowledge towards cervical cancer screening and associated factors among urban health extension workers at Addis Ababa, Ethiopia: facility based cross-sectional survey | doi: 10.1186/s12885-021-07952-z | Outcome of interest not available |
|  | Tesfahunei et al. | Human papillomavirus self-sampling versus standard clinician-sampling for cervical cancer screening in sub-Saharan Africa: a systematic review and meta-analysis of randomized controlled trials | doi: 10.1186/s13027-021-00380-5 | Outcome of interest not available |
|  | Lakew et al. | Cervical cancer screening practice and associated factors among female health care professionals in Ethiopia 2024: a systematic review and meta-analysis | doi: 10.1186/s12885-024-12743-3 | Outcome of interest not available |
|  | Fouw et al. | Local community networks in the fight against cervical cancer: the role of coffee ceremonies in the uptake of screening in Ethiopia | doi: 10.1177/0049475519864763. | Outcome of interest not available |
|  | Birhanu et al. | Health seeking behavior for cervical cancer in Ethiopia: a qualitative study | doi: 10.1186/1475-9276-11-83 | Qualitative |
|  | Bante et al. | Uptake of pre-cervical cancer screening and associated factors among reproductive age women in Debre Markos town, Northwest Ethiopia, 2017 | doi: 10.1186/s12889-019-7398-5 | Outcome of interest not available |
|  | Aynalem et al. | Utilization of cervical cancer screening and associated factors among women in Debremarkos town, Amhara region, Northwest Ethiopia: Community based cross-sectional study | doi: 10.1371/journal.pone.0231307 | Outcome of interest not available |
|  | Abu et al. | The role of health education on cervical cancer screening uptake at selected health centers in Addis Ababa | doi: 10.1371/journal.pone.0239580 | Outcome of interest not available |
|  | Tsegaye et al | Adherence and Correlates of Cervical Cancer Screening Among East African Immigrant Women in Washington State | doi: 10.1007/s40615-024-02038-5 | Outcome of interest not available |
|  | Chali et al. | Knowledge on cervical cancer, attitude toward its screening, and associated factors among reproductive age women in Metu Town, Ilu Aba Bor, South West Ethiopia, 2018: community-based cross-sectional study | doi: 10.1002/cnr2.1382 | Outcome of interest not available |
|  | Gebreegzabiher et al. | Cervical cancer screening and its associated factors among women of reproductive age in Kenya: further analysis of Kenyan demographic and health survey 2022 | doi: 10.1186/s12889-024-18148-y | Outcome of interest not available |
|  | Dulla et al. | Knowledge about cervical cancer screening and its practice among female health care workers in southern Ethiopia: a cross-sectional study | doi: 10.2147/IJWH.S132202 | Outcome of interest not available |
|  | Teame et al. | Factors affecting utilization of cervical cancer screening services among women attending public hospitals in Tigray region, Ethiopia, 2018; Case control study | doi: 10.1371/journal.pone.0213546 | Outcome of interest not available |
|  | Tadesse et al | Knowledge, Attitude, and Practice (KAP) toward Cervical Cancer Screening among Adama Science and Technology University Female Students, Ethiopia | doi: 10.1155/2022/2490327 | Outcome of interest not available |
|  | Mesafint et al. | Health Seeking Behavior of Patients Diagnosed with Cervical Cancer in Addis Ababa, Ethiopia | doi: 10.4314/ejhs.v28i2.2 | Outcome of interest not available |
|  | Melesse et al. | Utilization of cervical cancer screening service among female health workforces in public health institutions in south east Ethiopia, a cross-sectional study | doi: 10.1016/j.heliyon.2023.e23086 | Outcome of interest not available |
|  | Misgun et al. | Knowledge, practice of cervical cancer screening and associated factors among women police members of Addis Ababa police commission Ethiopia | doi: 10.1186/s12885-023-11478-x | Outcome of interest not available |
|  | Wondimagegnew et al. | Burden of Cancer and Utilization of Local Surgical Treatment Services in Rural Hospitals of Ethiopia: A Retrospective Assessment from 2014 to 2019 | doi: 10.1093/oncolo/oyac127 | Outcome of interest not available |
|  | Jede et al. | Home-based HPV self-sampling assisted by a cloud-based electronic data system: Lessons learnt from a pilot community cervical cancer screening campaign in rural Ethiopia | doi: 10.1016/j.pvr.2020.100198 | Outcome of interest not available |
|  | Fentie et al. | Factors affecting cervical cancer screening uptake, visual inspection with acetic acid positivity and its predictors among women attending cervical cancer screening service in Addis Ababa, Ethiopia | doi: 10.1186/s12905-020-01008-3 | Outcome of interest not available |
|  | Gemeda et al. | Prevalence and Predictor of Cervical Cancer Screening Service Uptake Among Women Aged 25 Years and Above in Sidama Zone, Southern Ethiopia, Using Health Belief Model | doi: 10.1177/1073274820954460 | Outcome of interest not available |
|  | Assefa et al. | Cervical cancer screening service utilization and associated factors among HIV positive women attending adult ART clinic in public health facilities, Hawassa town, Ethiopia: a cross-sectional study | doi: 10.1186/s12913-019-4718-5 | Outcome of interest not available |
|  | Brandt et al | Genital self-sampling for HPV-based cervical cancer screening: a qualitative study of preferences and barriers in rural Ethiopia | doi: 10.1186/s12889-019-7354-4 | Outcome of interest not available |
|  | Tesfaye et al. | Utilization of cervical cancer screening and determinant factors among female nurses in selected public hospitals in Addis Ababa, Ethiopia | doi: 10.1016/j.ctarc.2024.100815 | Outcome of interest not available |
|  | Yasin et al. | Cervical cancer screening service utilisation and related factors among women on antiretroviral therapy in public health facilities of Asella town, Ethiopia, cross-sectional study | doi: 10.1186/s12879-024-10003-0 | Outcome of interest not available |
|  | Solomon et al. | Predictors of cervical cancer screening practice among HIV positive women attending adult anti-retroviral treatment clinics in Bishoftu town, Ethiopia: the application of a health belief model | doi: 10.1186/s12885-019-6171-6 | Outcome of interest not available |
|  | Zegeye et al. | Uptake of cervical cancer screening and associated factors among HIV positive women attending adult art clinic at public hospitals in Addis Ababa, Ethiopia, 2022 | doi: 10.1186/s12905-024-03169-x | Outcome of interest not available |
|  | Atnafu et al. | Women's Satisfaction with Cervical Cancer Screening Services and Associated Factors in Maternal Health Clinics of Jimma Town Public Health Facilities, Southwest Ethiopia | doi: 10.2147/CMAR.S327369 | Outcome of interest not available |
|  | Wollancho et al. | Determining behavioral intention and its predictors towards cervical cancer screening among women in Gomma district, Jimma, Ethiopia: Application of the theory of planned behavior | doi: 10.1371/journal.pone.0238472 | Included |
|  | Bogale et al. | Knowledge and Practice of Women With HIV on Cervical Cancer Prevention and Control and their Attributes to Utilize the Screening Services in Ethiopia: A Cross Sectional Study | doi: 10.1177/10732748241284943. | Outcome of interest not available |
|  | Nigussie et al. | Knowledge Toward Cervical Cancer and Its Determinants Among Women Aged 30-49 in Jimma Town, Southwest Ethiopia | doi: 10.1177/1073274820983027 | Outcome of interest not available |
|  | Bekalu et al | Uptake of cervical cancer screening and associated factors among human immune virus positive women attending antiretroviral therapy clinic in public health facilities, Northeast Ethiopia, 2022: A cross-sectional study | doi: 10.1177/20503121231225935 | Outcome of interest not available |
|  | Endalew et al. | Knowledge and practice of cervical cancer screening and associated factors among reproductive age group women in districts of Gurage zone, Southern Ethiopia. A cross-sectional study | doi: 10.1371/journal.pone.0238869 | Outcome of interest not available |
|  | Shiferaw et al. | Knowledge about cervical cancer and barriers toward cervical cancer screening among HIV-positive women attending public health centers in Addis Ababa city, Ethiopia | doi: 10.1002/cam4.1334 | Outcome of interest not available |
|  | Hailemariam et al. | Magnitude and associated factors of VIA positive test results for cervical cancer screening among refugee women aged 25-49 years in North Ethiopia | doi: 10.1186/s12885-020-07344-9 | Outcome of interest not available |
|  | Argaw et al. | Knowledge, and practice of cervical cancer prevention and associated factors among commercial sex workers in Shashemene Town, West Arsi, Oromia Region, Ethiopia | doi: 10.1186/s12905-022-01819-6 | Outcome of interest not available |
|  | Gebregzabiher et al. | Correlates of cervical cancer screening uptake among female under graduate students of Aksum University, College of Health Sciences, Tigray, Ethiopia | doi: 10.1186/s13104-019-4570-z | Outcome of interest not available |
|  | Bogale et al. | Knowledge, attitude and practice of cervical cancer screening among women infected with HIV in Africa: Systematic review and meta-analysis | doi: 10.1371/journal.pone.0249960 | Review article |
|  | Merga et al. | Clients' satisfaction with cervical cancer screening services and influencing factors at public health facilities in Debre Markos town, Northwest Ethiopia, 2022/23: a convergent parallel mixed method | doi: 10.1186/s12905-024-03250-5 | Qualitative, different outcome |
|  | Teklehaimanot et al. | Precancerous lesion determinants in women attending cervical cancer screening at public health facilities in North Shoa Zone, Amhara, Ethiopia: an unmatched case-control study | doi: 10.1186/s12905-024-03113-z | Outcome of interest not available |
|  | Mekurya et al. | Prevalence of Cervical Cancer and Associated Factors Among Women Attended Cervical Cancer Screening Center at Gahandi Memorial Hospital, Ethiopia | doi: 10.1177/11769351211068431 | Outcome of interest not available |
|  | Deribie et al. | Histopathological profile of cervical punch biopsies and risk factors associated with high-grade cervical precancerous lesions and cancer in northwest Ethiopia | doi: 10.1371/journal.pone.0274466. | Outcome of interest not available |
|  | Tekle et al. | Knowledge, Attitude and Practice Towards Cervical Cancer Screening Among Women and Associated Factors in Hospitals of Wolaita Zone, Southern Ethiopia | doi: 10.2147/CMAR.S240364 | Outcome of interest not available |
|  | Tenkir et al. | The magnitude of precancerous cervical lesions and its associated factors among women screened for cervical cancer at a referral center in southern Ethiopia, 2021: a cross-sectional study | doi: 10.3389/fgwh.2023.1187916 | Outcome of interest not available |
|  | Beyene et al. | Risk factors for precancerous cervical lesion among women screened for cervical cancer in south Ethiopia: Unmatched case-control study | doi: 10.1371/journal.pone.0254663 | Outcome of interest not available |
|  | Geremew et al. | Comprehensive knowledge on cervical cancer, attitude towards its screening and associated factors among women aged 30-49 years in Finote Selam town, northwest Ethiopia | doi: 10.1186/s12978-018-0471-1 | Outcome of interest not available |
|  | Abamecha et al. | Psychographic predictors of intention to use cervical cancer screening services among women attending maternal and child health services in Southern Ethiopia: the theory of planned behavior (TPB) perspective | doi: 10.1186/s12889-019-6745-x | Outcome of interest not available |
|  | Tsegay et al. | Knowledge, Attitude, and Practice on Cervical Cancer Screening and Associated Factors Among Women Aged 15-49 Years in Adigrat Town, Northern Ethiopia, 2019: A Community-Based Cross-Sectional Study | doi: 10.2147/IJWH.S261204. | Outcome of interest not available |
|  | Aayana et al. | Effectiveness of couple education and counseling on uptake of cervical cancer screening among women in Southern Ethiopia: a cluster randomized trial | doi: 10.1038/s41598-024-61988-2 | Outcome of interest not available |
|  | Belete et al. | Willingness and acceptability of cervical cancer screening among women living with HIV/AIDS in Addis Ababa, Ethiopia: a cross sectional study | doi: 10.1186/s40661-015-0012-3. | Outcome of interest not available |
|  | Hussien et al. | Factors influencing the uptake and utilization of cervical cancer screening services among women attending public health centers in Addis Ababa, Ethiopia: mixed methods study | doi: 10.1186/s12905-023-02850-x | Outcome of interest not available |
|  | Assefa et al. | Utilization and associated factors of cervical cancer screening service among eligible women attending maternal health services at Adare General Hospital, Hawassa city, Southern Ethiopia | doi: 10.1038/s41598-024-52924-5 | Outcome of interest not available |
|  | Kantelhardt et al. | Cervical cancer in Ethiopia: survival of 1,059 patients who received oncologic therapy | doi: 10.1634/theoncologist.2013-0326 | Outcome of interest not available |
|  | Habtamu et al. | Uptake of Cervical Cancer Screening and Its Barriers Using Health Belief Model Among Health Professionals Working in Public Hospitals in South Gondar Zone, Northcentral Ethiopia: Multicenter Cross-Sectional Study | doi: 10.1089/whr.2023.0030 | Outcome of interest not available |
|  | Aytenew et al. | Uptake of Cervical Cancer Screening and Its Barriers Using Health Belief Model Among Health Professionals Working in Public Hospitals in South Gondar Zone, Northcentral Ethiopia: Multicenter Cross-Sectional Study | doi: 10.1089/whr.2023.0030 | Outcome of interest not available |
|  | Mathivanan et al. | Enhancing cervical cancer detection and robust classification through a fusion of deep learning models | doi: 10.1038/s41598-024-61063-w | Outcome of interest not available |
|  | Atnafu et al. | Drivers of cervical cancer prevention and management in sub-Saharan Africa: a qualitative synthesis of mixed studies | doi: 10.1186/s12961-023-01094-3 | Outcome of interest not available |
|  | Hussien et al. | Assessing the influence of the health system on access to cervical cancer prevention, screening, and treatment services at public health centers in Addis Ababa, Ethiopia | doi: 10.1371/journal.pone.0300152 | Outcome of interest not available |
|  | Tadesse et al. | Knowledge, Attitude, and Practice (KAP) toward Cervical Cancer Screening among Adama Science and Technology University Female Students, Ethiopia | https://doi.org/10.1155/2022/2490327 | Outcome of interest not available |
|  | Getachew et al. | Cervical cancer screening knowledge and barriers among women in Addis Ababa, Ethiopia | https://doi.org/10.1371/journal.pone.0216522 | Outcome of interest not available |
|  | Ruddies et al. | Cervical cancer screening in rural Ethiopia: a cross- sectional knowledge, attitude and practice study | https://doi.org/10.1186/s12885-020-07060-4 | Outcome of interest not available |
|  | Tekle et al. | Knowledge, Attitude and Practice Towards Cervical Cancer Screening Among Women and Associated Factors in Hospitals of Wolaita Zone, Southern Ethiopia | https://doi.org/10.2147/CMAR.S240364 | Outcome of interest not available |
|  | Tilahun et al. | Knowledge, attitude and practice of cervical cancer screening and associated factors amongst female students at Wollega University, western Ethiopia | https://doi.org/10.1186/s13104-019-4564-x | Outcome of interest not available |
|  | Bayu et al. | Cervical Cancer Screening Service Uptake and Associated Factors among Age Eligible Women in Mekelle Zone, Northern Ethiopia, 2015: A Community Based Study Using Health Belief Model | https://doi.org/10.1371/journal.pone.0149908 | Outcome of interest not available |
|  | Ayenew et al. | Uptake of cervical cancer screening service and associated factors among age-eligible women in Ethiopia: systematic review and meta-analysis | https://doi.org/10.1186/s13027-020-00334-3 | Review article |
|  | Yimer et al. | Cervical cancer screening uptake in Sub-Saharan Africa: a systematic review and meta-analysis | https://doi.org/10.1016/j.puhe.2021.04.014 | Review article |
|  | Aynalem et al. | Utilization of cervical cancer screening and associated factors among women in Debremarkos town, Amhara region, Northwest Ethiopia: Community based cross-sectional study | https://doi.org/10.1371/journal.pone.0231307 | Outcome of interest not available |
|  | Gebru et al. | Utilization of Cervical Carcinoma Screening Service and Associated Factors among Currently Married Women in Arba Minch Town, Southern Ethiopia. | http://10.140.5.162//handle/123456789/2471 | Outcome of interest not available |
|  | Nigussie | Cervical cancer screening service utilization and associated factors among age-eligible women in Jimma town using health belief model, South West Ethiopia | https://doi.org/10.1186/s12905-019-0826-y | Outcome of interest not available |
|  | Teame et al. | Factors affecting utilization of cervical cancer screening services among women attending public hospitals in Tigray region, Ethiopia, 2018; Case control study | https://doi.org/10.1371/journal.pone.0213546 | Outcome of interest not available |
|  | Geremew, A.B., et al. | Comprehensive knowledge on cervical cancer, attitude towards its screening and associated factors among women aged 30–49 years in Finote Selam town, northwest Ethiopia | https://doi.org/10.1186/s12978-018-0471-1 | Outcome of interest not available |
|  | Kassie et al. | Impact of knowledge and attitude on the utilization rate of cervical cancer screening tests among Ethiopian women: A systematic review and meta-analysis | https://doi.org/10.1371/journal.pone.0239927 | Review article |
|  | Endalew et al. | Knowledge and practice of cervical cancer screening and associated factors among reproductive age group women in districts of Gurage zone, Southern Ethiopia. A cross-sectional study | https://doi.org/10.1371/journal.pone.0238869 | Outcome of interest not available |
|  | Kress et al. | Knowledge, attitudes, and practices regarding cervical cancer and screening among Ethiopian health care workers | https://doi.org/10.2147/IJWH.S85138 | Outcome of interest not available |
|  | Fentie, A.M. et al. | Factors affecting cervical cancer screening uptake, visual inspection with acetic acid positivity and its predictors among women attending cervical cancer screening service in Addis Ababa, Ethiopia | https://doi.org/10.1186/s12905-020-01008-3 | Outcome of interest not available |
|  | Bante et al. | Uptake of pre-cervical cancer screening and associated factors among reproductive age women in Debre Markos town, Northwest Ethiopia, 2017 | https://doi.org/10.1186/s12889-019-7398-5 | Outcome of interest not available |
|  | Dulla et al. | Knowledge about cervical cancer screening and its practice among female health care workers in southern Ethiopia: a cross-sectional study | doi/full/10.2147/IJWH.S132202#d1e117 | Outcome of interest not available |
|  | Kasim et al | Cervical Cancer Screening Service Utilization and Associated Factors among Women in the Shabadino District, Southern Ethiopia | https://doi.org/10.1155/2020/6398394 | Outcome of interest not available |
|  | Getahun, F. et al | Comprehensive knowledge about cervical cancer is low among women in Northwest Ethiopia | https://doi.org/10.1186/1471-2407-13-2 | Outcome of interest not available |
|  | Bedell et al. | Cervical Cancer Screening: Past, Present, and Future | https://doi.org/10.1016/j.sxmr.2019.09.005 | Outcome of interest not available |
|  | Abu et al. | The role of health education on cervical cancer screening uptake at selected health centers in Addis Ababa | https://doi.org/10.1371/journal.pone.0239580 | Outcome of interest not available |
|  | Perkins et al. | Cervical Cancer Screening  A Review | doi:10.1001/jama.2023.13174 | Review |
|  | Birhanu, Z., et al. | Health seeking behavior for cervical cancer in Ethiopia: a qualitative study | https://doi.org/10.1186/1475-9276-11-83 | Outcome of interest not available |
|  | Fylan et al. | Screening for cervical cancer: a review of women's attitudes, knowledge, and behaviour. | https://bjgp.org/content/48/433/1509.short | Outcome of interest not available |
|  | Gatumo, M., et al. | Women’s knowledge and attitudes related to cervical cancer and cervical cancer screening in Isiolo and Tharaka Nithi counties, Kenya: a cross-sectional study | https://doi.org/10.1186/s12885-018-4642-9 | Outcome of interest not available |
|  | Denny et al. | Screening for cervical cancer in developing countries | https://doi.org/10.1016/j.vaccine.2006.05.121 | Outcome of interest not available |
|  | Shiferaw et al. | Knowledge about cervical cancer and barriers toward cervical cancer screening among HIV-positive women attending public health centers in Addis Ababa city, Ethiopia | https://doi.org/10.1002/cam4.1334 | Outcome of interest not available |
|  | S A ahmed et al. | Knowledge, attitude and practice of cervical cancer screening among market women in Zaria | https://doi.org/10.4103/0300-1652.122337 | Outcome of interest not available |
|  | Taneja et al. | Knowledge, Attitude, and Practice on Cervical Cancer and Screening Among Women: A Review | https://doi.org/10.1177/10732748211010799 | Review |
|  | BU Ezem et al. | Awareness and Uptake of Cervical Cancer Screening in Owerri | DOI: 10.4103/1596-3519.55727 | Outcome of interest not available |
|  | Goldie et al. | Cost-Effectiveness of Cervical-Cancer Screening in Five Developing Countries | DOI: 10.1056/NEJMsa044278 | Outcome of interest not available |
|  | Deribie et al. | Histopathological profile of cervical punch biopsies and risk factors associated with high-grade cervical precancerous lesions and cancer in northwest Ethiopia | doi: 10.1371/journal.pone.0274466. | Outcome of interest not available |
|  | Teame et al. | Factors affecting utilization of cervical cancer screening services among women attending public hospitals in Tigray region, Ethiopia, 2018; Case control study | doi: 10.1371/journal.pone.0213546 | Outcome of interest not available |
|  | Tadesse et al | Knowledge, Attitude, and Practice (KAP) toward Cervical Cancer Screening among Adama Science and Technology University Female Students, Ethiopia | doi: 10.1155/2022/2490327 | Outcome of interest not available |
|  | Endale et al. | MiRNA in cervical cancer: Diagnosis to therapy: Systematic review | doi: 10.1016/j.heliyon.2024.e24398. | Review |
|  | Amado et al. | Cervical cancer screening practices and its associated factors among females of reproductive age in Durame town, Southern Ethiopia | doi: 10.1371/journal.pone.0279870. | Different outcome |
|  | Biazin et al. | Concordance of Anyplex™ II HPV HR assays with reference HPV assays in cervical cancer screening: Systematic review | doi: 10.1016/j.jviromet.2021 | Review |
|  | Mohamed et al. | Cervical Cancer Screening Service Utilization and Associated Factors Among Women Living With HIV Receiving Anti-Retroviral Therapy at Adama Hospital Medical College, Ethiopia | doi: 10.1177/23779608231152072. | Outcome of interest not available |
|  | Tsegaye et al. | Evaluation of cervical cancer screening program in Gondar city administration public health facilities, Northwest Ethiopia, 2021: mixed method approach | doi: 10.1186/s12885-023-11533-7. | Outcome of interest not available |
|  | Peirson, L., | Screening for cervical cancer: a systematic review and meta-analysis | https://doi.org/10.1186/2046-4053-2-35 | Review |
|  | Zhang et al. | Cervical cancer: Epidemiology, risk factors and screening | https://doi.org/10.21147/j.issn.1000-9604.2020.06.05 | Different outcome |
|  | Kassa et al. | Survival of women with cervical cancer in East Africa: a systematic review and meta-analysis | doi: 10.1080/01443615.2023.225330. | Review |
|  | Gizaw et al. | Uptake of Cervical Cancer Screening in Ethiopia by Self-Sampling HPV DNA Compared to Visual Inspection with Acetic Acid: A Cluster Randomized Trial | doi: 10.1158/1940-6207.CAPR-19-0156. | Outcome of interest not available |
|  | Luito et al. | Impact of health education intervention on knowledge and perception of cervical cancer and cervical screening uptake among adult women in rural communities in Nigeria | doi: 10.1186/s12905-023-02479-w. | Outcome of interest not available |
|  | Legasu et al. | Determinants of cervical cancer screening service utilization among women attending healthcare services in Amhara region referral hospitals: a case-control study | doi: 10.1186/s12905-022-02071-8 | Outcome of interest not available |
|  | M Urasa | Knowledge of cervical cancer and screening practices of nurses at a regional hospital in Tanzania | https://www.ajol.info/index.php/ahs/article/view/64992 | Different outcome |
|  | Mengesha et al. | Uptake and barriers to cervical cancer screening among human immunodeficiency virus-positive women in Sub Saharan Africa: a systematic review and meta-analysis | doi: 10.1186/s12905-023-02479-w. | Review |
|  | Xinhu et al. | Review of the Cervical Cancer Burden and Population-Based Cervical Cancer Screening in China | https://journal.waocp.org/article_31598.html | Outcome of interest not available |
|  | Nakasige et al. | Cervical cancer screening and treatment in Uganda | https://doi.org/10.1016/j.gore.2017.01.009 | Outcome of interest not available |
|  | Mitiku et al. | Knowledge about Cervical Cancer and Associated Factors among 15-49 Year Old Women in Dessie Town, Northeast Ethiopia | https://doi.org/10.1371/journal.pone.0163136 | Outcome of interest not available |
|  | Endalew et al. | Knowledge and practice of cervical cancer screening and associated factors among reproductive age group women in districts of Gurage zone, Southern Ethiopia. A cross-sectional study | doi: 10.1371/journal.pone.0238869 | Outcome of interest not available |
|  | Shiferaw et al. | Knowledge about cervical cancer and barriers toward cervical cancer screening among HIV-positive women attending public health centers in Addis Ababa city, Ethiopia | doi: 10.1002/cam4.1334 | Outcome of interest not available |
|  | Okunowo et al. | Women's knowledge of cervical cancer and uptake of Pap smear testing and the factors influencing it in a Nigerian tertiary hospital | https://doi.org/10.1016/j.jcrpr.2018.02.001 | Outcome of interest not available |
|  | Meer et al. | Knowledge, attitude and practices regarding cervical cancer and screening among women visiting primary health care in Qatar | https://iris.who.int/handle/10665/118198 | Outcome of interest not available |
|  | Jia et al. | Knowledge about Cervical Cancer and Barriers of Screening Program among Women in Wufeng County, a High-Incidence Region of Cervical Cancer in China | https://doi.org/10.1371/journal.pone.0067005 | Outcome of interest not available |
|  | Ararsa et al. | Knowledge towards cervical cancer screening and associated factors among urban health extension workers at Addis Ababa, Ethiopia: facility based cross-sectional survey | doi: 10.1186/s12885-021-07952-z | Outcome of interest not available |
|  | Tesfahunei et al. | Human papillomavirus self-sampling versus standard clinician-sampling for cervical cancer screening in sub-Saharan Africa: a systematic review and meta-analysis of randomized controlled trials | doi: 10.1186/s13027-021-00380-5 | Different outcome |
|  | Lakew et al. | Cervical cancer screening practice and associated factors among female health care professionals in Ethiopia 2024: a systematic review and meta-analysis | doi: 10.1186/s12885-024-12743-3 | Review article |
|  | Abytenew et al. | Uptake of Cervical Cancer Screening and Its Barriers Using Health Belief Model Among Health Professionals Working in Public Hospitals in South Gondar Zone, Northcentral Ethiopia: Multicenter Cross-Sectional Study | doi: 10.1089/whr.2023.0030 | Outcome of interest not available |
|  | Mathivanan et al. | Enhancing cervical cancer detection and robust classification through a fusion of deep learning models | doi: 10.1038/s41598-024-61063-w | Outcome of interest not available |
|  | Write jr et al. | Alternative approaches to cervical cancer screening for developing countries | https://doi.org/10.1016/j.bpobgyn.2011.11.004 | Outcome of interest not available |
|  | Cronje et al. | Screening for cervical cancer in developing countries | https://doi.org/10.1016/j.ijgo.2003.09.009 | Outcome of interest not available |
|  | Insinga et al. | Diagnoses and outcomes in cervical cancer screening: A population-based study | https://doi.org/10.1016/j.ajog.2004.01.043 | Outcome of interest not available |
|  | Endalew et al. | Knowledge and practice of cervical cancer screening and associated factors among reproductive age group women in districts of Gurage zone, Southern Ethiopia. A cross-sectional study | https://doi.org/10.1371/journal.pone.0238869 | Outcome of interest not available |
|  | Mitiku et al. | Knowledge about Cervical Cancer and Associated Factors among 15-49 Year Old Women in Dessie Town, Northeast Ethiopia | https://doi.org/10.1371/journal.pone.0163136 | Outcome of interest not available |
|  | Kasa et al. | Knowledge, attitude and practice towards cervical cancer among women in Finote Selam city administration, West Gojjam Zone, Amhara Region, North West Ethiopia, 2017 | https://doi.org/10.4314/ahs.v18i3.20 | Outcome of interest not available |
|  | Nakasi et al. | Cervical cancer screening and treatment in Uganda | https://doi.org/10.1016/j.gore.2017.01.009 | Outcome of interest not available |
|  | Mutyaba, T., et al. | Knowledge, attitudes and practices on cervical cancer screening among the medical workers of Mulago Hospital, Uganda | https://doi.org/10.1186/1472-6920-6-13 | Outcome of interest not available |
|  | Girma et al. | Cervical cancer screening practices and its associated factors among females of reproductive age in Durame town, Southern Ethiopia | https://doi.org/10.1371/journal.pone.0279870 | Outcome of interest not available |
|  | Ayenew, A.A., et al. | Uptake of cervical cancer screening service and associated factors among age-eligible women in Ethiopia: systematic review and meta-analysis | https://doi.org/10.1186/s13027-020-00334-3 | Review |
|  | Sayih et al. | Cervical cancer screening utilization and its predictors among women in bench Sheko Zone, Southwest Ethiopia: using health belief model | https://doi.org/10.1186/s12885-023-10927-x | Outcome of interest not available |
|  | Winta et al. | Utilization of cervical cancer screening and determinant factors among female nurses in selected public hospitals in Addis Ababa, Ethiopia | https://doi.org/10.1016/j.ctarc.2024.100815 | Outcome of interest not available |
|  | Abebaw et al. | Knowledge and practice of cervical cancer screening and associated factors among reproductive age group women in districts of Gurage zone, Southern Ethiopia. A cross-sectional study | doi: 10.1371/journal.pone.0238869 | Outcome of interest not available |
|  | Shimelis et al. | Knowledge about cervical cancer and barriers toward cervical cancer screening among HIV-positive women attending public health centers in Addis Ababa city, Ethiopia | doi: 10.1002/cam4.1334 | Outcome of interest not available |
|  | Haile et al. | Magnitude and associated factors of VIA positive test results for cervical cancer screening among refugee women aged 25-49 years in North Ethiopia | doi: 10.1186/s12885-020-07344-9 | Outcome of interest not available |
|  | Areawi et al. | Knowledge, and practice of cervical cancer prevention and associated factors among commercial sex workers in Shashemene Town, West Arsi, Oromia Region, Ethiopia | doi: 10.1186/s12905-022-01819-6 | Outcome of interest not available |
|  | Gebre et al. | Correlates of cervical cancer screening uptake among female under graduate students of Aksum University, College of Health Sciences, Tigray, Ethiopia | doi: 10.1186/s13104-019-4570-z | Outcome of interest not available |
|  | Belete et al. | Knowledge, attitude and practice of cervical cancer screening among women infected with HIV in Africa: Systematic review and meta-analysis | doi: 10.1371/journal.pone.0249960 | Review |
|  | Merga et al. | Clients' satisfaction with cervical cancer screening services and influencing factors at public health facilities in Debre Markos town, Northwest Ethiopia, 2022/23: a convergent parallel mixed method | doi: 10.1186/s12905-024-03250-5 | Outcome of interest not available |
|  | Teketel et al. | Precancerous lesion determinants in women attending cervical cancer screening at public health facilities in North Shoa Zone, Amhara, Ethiopia: an unmatched case-control study | doi: 10.1186/s12905-024-03113-z | Outcome of interest not available |
|  | Fuzli et al. | Cervical cancer screening in the United States: Challenges and potential solutions for underscreened groups | https://doi.org/10.1016/j.ypmed.2020.106400 | Outcome of interest not available |
|  | Bansal et al. | Knowledge, attitude, and practices related to cervical cancer among adult women: A hospital-based cross-sectional study | https://doi.org/10.4103/0976-9668.159993 | Outcome of interest not available |
|  | Legasuni et al. | Determinants of cervical cancer screening service utilization among women attending healthcare services in Amhara region referral hospitals: a case-control study | doi: 10.1186/s12905-022-02071-8 | Outcome of interest not available |
|  | Tesfayesus et al. | Cervical cancer screening practice and associated factors among women employees in Wolaita Zone hospitals, Southern Ethiopia, 2017: cross-sectional study | doi: 10.11604/pamj.2022.42.318.34675 | Outcome of interest not available |
|  | Sharon et al. | Cervical cancer screening utilization and associated factors among women attending antenatal care at Asella Referral and Teaching Hospital, Arsi zone, South Central Ethiopia | doi: 10.1186/s12905-023-02326-y. | Outcome of interest not available |
|  | Endalemaw et al. | MiRNA in cervical cancer: Diagnosis to therapy: Systematic review | doi: 10.1016/j.heliyon.2024.e24398. | Outcome of interest not available |
|  | Aman et al. | Cervical cancer screening practices and its associated factors among females of reproductive age in Durame town, Southern Ethiopia | doi: 10.1371/journal.pone.0279870. | Outcome of interest not available |
|  | Bixuneh et al. | Concordance of Anyplex™ II HPV HR assays with reference HPV assays in cervical cancer screening: Systematic review | doi: 10.1016/j.jviromet.2021 | Review |
|  | Mohider et al. | Cervical Cancer Screening Service Utilization and Associated Factors Among Women Living With HIV Receiving Anti-Retroviral Therapy at Adama Hospital Medical College, Ethiopia | doi: 10.1177/23779608231152072. | Outcome of interest not available |
|  | Erku, D.A., et al. | Comprehensive knowledge and uptake of cervical cancer screening is low among women living with HIV/AIDS in Northwest Ethiopia | https://doi.org/10.1186/s40661-017-0057-6 | Outcome of interest not available |
|  | Lyimo, F.S., et al. | Demographic, knowledge, attitudinal, and accessibility factors associated with uptake of cervical cancer screening among women in a rural district of Tanzania: Three public policy implications | https://doi.org/10.1186/1471-2458-12-22 | Outcome of interest not available |
|  | Getasew et al. | Cervical cancer screening "see and treat approach": real-life uptake after invitation and associated factors at health facilities in Gondar, Northwest Ethiopia | doi: 10.1186/s12885-021-08761-0. | Outcome of interest not available |
|  | Chungar et al. | Socioeconomic inequalities in cervical precancer screening among women in Ethiopia, Malawi, Rwanda, Tanzania, Zambia and Zimbabwe: analysis of Population-Based HIV Impact Assessment surveys | doi: 10.1136/bmjopen-2022-067948. | Outcome of interest not available |
|  | Belayneh et al. | Cervical Cancer Screening Utilization and Associated Factors Among Women Aged 30 to 49 Years in Dire Dawa, Eastern Ethiopia | doi: 10.1177/1073274820958701. | Outcome of interest not available |
|  | Byantu et al. | Women's and health professionals' perceptions, beliefs and barriers to cervical cancer screening uptake in Southern Ethiopia: a qualitative study | doi: 10.1080/26410397.2023.2258477. | Qualitative study |
|  | Peter A Aboyeji et al. | Knowledge, Attitude and Practice of Cervical Smear as a Screening Procedure for Cervical Cancer in Ilorin, Nigeria | https://doi.org/10.4314/tjog.v21i2.14482 | Outcome of interest not available |
|  | Ebissa, N. I., et al. | Knowledge, practice, and barriers toward cervical cancer screening in Elmina, Southern Ghana | https://doi.org/10.2147/IJWH.S71797 | Outcome of interest not available |
|  | Mamo et al. | Usage of cervical cancer screening services among HIV-positive women in Southern Ethiopia: a multicentre cross-sectional study | doi: 10.1136/bmjopen-2022-068253. | Outcome of interest not available |
|  | Asse et al. | Determinants of cervical cancer screening utilization among women attending health facilities of Dessie town, Northeast Ethiopia | doi: 10.1186/s12885-022-10447-0. | Outcome of interest not available |
|  | Nedejjo et al. | Uptake of Cervical Cancer Screening and Associated Factors among Women in Rural Uganda: A Cross Sectional Study | https://doi.org/10.1371/journal.pone.0149696 | Outcome of interest not available |
|  | Abe et al. | Knowledge and practice of cervical cancer screening and associated factors among reproductive age group women in districts of Gurage zone, Southern Ethiopia. A cross-sectional study | doi: 10.1371/journal.pone.0238869 | Outcome of interest not available |
|  | Hailu, A., et al. | Patient side cost and its predictors for cervical cancer in Ethiopia: a cross sectional hospital based study | https://doi.org/10.1186/1471-2407-13-69 | Outcome of interest not available |
|  | Misgana et al. | Knowledge, practice of cervical cancer screening and associated factors among women police members of Addis Ababa police commission Ethiopia | doi: 10.1186/s12885-023-11478-x | Outcome of interest not available |
|  | Wondi et al. | Burden of Cancer and Utilization of Local Surgical Treatment Services in Rural Hospitals of Ethiopia: A Retrospective Assessment from 2014 to 2019 | doi: 10.1093/oncolo/oyac127 | Outcome of interest not available |
|  | Gedelu et al. | Home-based HPV self-sampling assisted by a cloud-based electronic data system: Lessons learnt from a pilot community cervical cancer screening campaign in rural Ethiopia | doi: 10.1016/j.pvr.2020.100198 | Outcome of interest not available |
|  | Fanontie et al. | Factors affecting cervical cancer screening uptake, visual inspection with acetic acid positivity and its predictors among women attending cervical cancer screening service in Addis Ababa, Ethiopia | doi: 10.1186/s12905-020-01008-3 | Outcome of interest not available |
|  | Gammeda et al. | Prevalence and Predictor of Cervical Cancer Screening Service Uptake Among Women Aged 25 Years and Above in Sidama Zone, Southern Ethiopia, Using Health Belief Model | doi: 10.1177/1073274820954460 | Outcome of interest not available |
|  | Ndikom, C.M., et al. | Awareness, perception and factors affecting utilization of cervical cancer screening services among women in Ibadan, Nigeria: a qualitative stud | https://doi.org/10.1186/1742-4755-9-11 | Outcome of interest not available |
|  | Enferm et al | Cervical cancer: knowledge, attitude and practice on the prevention examination | https://doi.org/10.1590/0034-7167-2017-0645 | Outcome of interest not available |
|  | Gibat et al. | Less known but greatly feared: Cervical cancer in Ethiopia community awareness | doi: 10.1016/j.heliyon.2024.e28328. | Outcome of interest not available |
|  | Luel et al. | Factors affecting cervical cancer screening service uptake among clients visiting the ART clinic at Hawassa University Comprehensive Specialized Hospital, Hawassa, Ethiopia: A cross-sectional study | doi: 10.1016/j.jcpo.2023.100422. | Outcome of interest not available |
|  | Tesfera et al. | Uptake of Cervical Cancer Screening and Associated Factors Among 15-49-Year-Old Women in Dessie Town, Northeast Ethiopia | doi: 10.1007/s13187-016-1021-6. | Outcome of interest not available |
|  | Selam et al. | Knowledge and awareness of cervical cancer in Southwestern Ethiopia is lacking: A descriptive analysis | doi: 10.1371/journal.pone.0215117. | Outcome of interest not available |
|  | Aeyan et al. | Effectiveness of couple education and counseling on knowledge, attitude and uptake of cervical cancer screening service among women of child bearing age in Southern Ethiopia: A cluster randomized trial protocol | doi: 10.1371/journal.pone.0270663. | Outcome of interest not available |
|  | Ermiru et al. | Role of awareness on cervical cancer screening uptake among HIV positive women in Addis Ababa, Ethiopia: A cross-sectional study | doi: 10.1177/17455065211017041. | Outcome of interest not available |
|  | Destaye et al. | Cervical cancer screening "see and treat approach": real-life uptake after invitation and associated factors at health facilities in Gondar, Northwest Ethiopia | doi: 10.1186/s12885-021-08761-0. | Outcome of interest not available |
|  | Peto, Julian et al. | The cervical cancer epidemic that screening has prevented | https://www.thelancet.com/journals/lancet/article/PIIS0140-6736(04)16674-9/abstract | Outcome of interest not available |
|  | Kivistik, A., et al. | Women's knowledge about cervical cancer risk factors, screening, and reasons for non-participation in cervical cancer screening programme in Estonia | https://doi.org/10.1186/1472-6874-11-43 | Outcome of interest not available |
|  | Leimu et al | Barriers to utilisation of cervical cancer screening in Sub Sahara Africa | https://doi.org/10.1111/ecc.12444 | Outcome of interest not available |
|  | Mengesha, A., et al. | Knowledge and attitude towards cervical cancer among reproductive age group women in Gondar town, North West Ethiopia | https://doi.org/10.1186/s12889-020-8229-4 | Outcome of interest not available |
|  | Debebe et al. | Histopathological profile of cervical punch biopsies and risk factors associated with high-grade cervical precancerous lesions and cancer in northwest Ethiopia | doi: 10.1371/journal.pone.0274466. | Outcome of interest not available |
|  | Tamne et al. | Factors affecting utilization of cervical cancer screening services among women attending public hospitals in Tigray region, Ethiopia, 2018; Case control study | doi: 10.1371/journal.pone.0213546 | Outcome of interest not available |
|  | Birtukan et al. | Uptake of pre-cervical cancer screening and associated factors among reproductive age women in Debre Markos town, Northwest Ethiopia, 2017 | https://doi.org/10.1186/s12889-019-7398-5 | Outcome of interest not available |
|  | Duyea et al. | Knowledge about cervical cancer screening and its practice among female health care workers in southern Ethiopia: a cross-sectional study | doi/full/10.2147/IJWH.S132202#d1e117 | Outcome of interest not available |
|  | Kasimas et al | Cervical Cancer Screening Service Utilization and Associated Factors among Women in the Shabadino District, Southern Ethiopia | https://doi.org/10.1155/2020/6398394 | Outcome of interest not available |
|  | Fetahun, F. | Comprehensive knowledge about cervical cancer is low among women in Northwest Ethiopia | https://doi.org/10.1186/1471-2407-13-2 | Outcome of interest not available |
|  | Ebu, N.I., et al | Impact of health education intervention on knowledge and perception of cervical cancer and screening for women in Ghana | https://doi.org/10.1186/s12889-019-7867-x | Outcome of interest not available |
|  | Eze, Justus N. et al | Cervical cancer awareness and cervical screening uptake at the Mater Misericordiae Hospital, Afikpo, Southeast Nigeria | DOI: 10.4103/1596-3519.102856 | Outcome of interest not available |
|  | Ayenew, et al. | Uptake of cervical cancer screening service and associated factors among age-eligible women in Ethiopia: systematic review and meta-analysis | https://doi.org/10.1186/s13027-020-00334-3 | Review article |
|  | Jayih et al. | Cervical cancer screening utilization and its predictors among women in bench Sheko Zone, Southwest Ethiopia: using health belief model | https://doi.org/10.1186/s12885-023-10927-x | Outcome of interest not available |
|  | Fewanko et al. | Knowledge attitudes and practices of cervical cancer screening among urban and rural Nigerian women: a call for education and mass screening | https://doi.org/10.1111/j.1365-2354.2009.01175.x | Outcome of interest not available |
|  | Idowu et al. | Determinants of Cervical Cancer Screening Uptake among Women in Ilorin, North Central Nigeria: A Community-Based Study | https://doi.org/10.1155/2016/6469240 | Outcome of interest not available |
|  | Lalem et al. | Cervical cancer screening practice and associated factors among female health care professionals in Ethiopia 2024: a systematic review and meta-analysis | doi: 10.1186/s12885-024-12743-3 | Review article |
|  | Mouw et al. | Local community networks in the fight against cervical cancer: the role of coffee ceremonies in the uptake of screening in Ethiopia | doi: 10.1177/0049475519864763. | Different outcome |
|  | Birhanesh et al. | Health seeking behavior for cervical cancer in Ethiopia: a qualitative study | doi: 10.1186/1475-9276-11-83 | Qualitative |
|  | Bantayehu et al. | Uptake of pre-cervical cancer screening and associated factors among reproductive age women in Debre Markos town, Northwest Ethiopia, 2017 | doi: 10.1186/s12889-019-7398-5 | Outcome of interest not available |
|  | Catarino et al. | Cervical cancer screening in developing countries at a crossroad: Emerging technologies and policy choices | https://doi.org/10.5306/wjco.v6.i6.281 | Outcome of interest not available |
|  | Dereje et al. | Knowledge and acceptance of HPV vaccination and its associated factors among parents of daughters in Addis Ababa, Ethiopia: a community-based cross-sectional study. | 10.1186/s13027-021-00399-8 | Outcome of interest not available |
|  | Teferi R | Human Papillomavirus Knowledge, Perception, and Willingness to Receive Vaccination Among Female University Students in Addis Ababa University, Ethiopia,2022:a Mixed Method | http://etd.aau.edu.et/handle/123456789/32244 | Outcome of interest not available |
|  | Owoeye et al. | Knowledge and attitude towards cervical cancer screening among female students and staff in a tertiary institution in the Niger Delta | https://www.ajol.info/index.php/ijmbr/article/view/91935 | Outcome of interest not available |
|  | Mikrch et al. | Enhancing cervical cancer detection and robust classification through a fusion of deep learning models | doi: 10.1038/s41598-024-61063-w | Outcome of interest not available |
|  | Atrisaw et al. | Drivers of cervical cancer prevention and management in sub-Saharan Africa: a qualitative synthesis of mixed studies | doi: 10.1186/s12961-023-01094-3 | Different outcome |
|  | Ayenachew, et al. | Uptake of cervical cancer screening service and associated factors among age-eligible women in Ethiopia: systematic review and meta-analysis | https://doi.org/10.1186/s13027-020-00334-3 | Review |
|  | Shayih et al. | Cervical cancer screening utilization and its predictors among women in bench Sheko Zone, Southwest Ethiopia: using health belief model | https://doi.org/10.1186/s12885-023-10927-x | Outcome of interest not available |
|  | Waintay et al. | Utilization of cervical cancer screening and determinant factors among female nurses in selected public hospitals in Addis Ababa, Ethiopia | https://doi.org/10.1016/j.ctarc.2024.100815 | Outcome of interest not available |
|  | Asfaw et al. | Utilization and associated factors of cervical cancer screening service among eligible women attending maternal health services at Adare General Hospital, Hawassa city, Southern Ethiopia | doi: 10.1038/s41598-024-52924-5 | Outcome of interest not available |
|  | Kante et al. | Cervical cancer in Ethiopia: survival of 1,059 patients who received oncologic therapy | doi: 10.1634/theoncologist.2013-0326 | Outcome of interest not available |
|  | Neatae et al. | Cervical cancer screening uptake and determinant factors among women in Ambo town, Western Oromia, Ethiopia: Community-based cross-sectional study | doi: 10.1002/cam4.4369. Epub 2021 Oct 27. | Outcome of interest not available |
|  | Teasfaye et al. | Cervical cancer screening uptake and associated factors among Women Living with Human Immunodeficiency Virus in public hospitals, eastern Ethiopia | doi: 10.3389/fonc.2023.1249151. eCollection 2023. | Outcome of interest not available |
|  | Zeawdie et al | Determinants of late-stage cervical cancer presentation in Ethiopia: a systematic review and meta-analysis | doi: 10.1186/s12885-023-11728-y. | Review |
|  | Asshenafi et al. | Characteristics of Women Seeking Cervical Cancer Cytology Screening in a Private Health Facility | doi: 10.3390/medicina59091624. | Outcome of interest not available |
|  | Zaaegeye et al. | Uptake of cervical cancer screening and associated factors among HIV positive women attending adult art clinic at public hospitals in Addis Ababa, Ethiopia, 2022 | doi: 10.1186/s12905-024-03169-x | Outcome of interest not available |
|  | Atnafu et al. | Women's Satisfaction with Cervical Cancer Screening Services and Associated Factors in Maternal Health Clinics of Jimma Town Public Health Facilities, Southwest Ethiopia | doi: 10.2147/CMAR.S327369 | Outcome of interest not available |
|  | Weollancho et al. | Determining behavioral intention and its predictors towards cervical cancer screening among women in Gomma district, Jimma, Ethiopia: Application of the theory of planned behavior | doi: 10.1371/journal.pone.0238472 | Outcome of interest not available |
|  | Baogale et al. | Knowledge and Practice of Women With HIV on Cervical Cancer Prevention and Control and their Attributes to Utilize the Screening Services in Ethiopia: A Cross Sectional Study | doi: 10.1177/10732748241284943. | Outcome of interest not available |
|  | Findalew et al. | Knowledge and practice of cervical cancer screening and associated factors among reproductive age group women in districts of Gurage zone, Southern Ethiopia. A cross-sectional study | doi: 10.1371/journal.pone.0238869 | Outcome of interest not available |
|  | Seahiferaw et al. | Knowledge about cervical cancer and barriers toward cervical cancer screening among HIV-positive women attending public health centers in Addis Ababa city, Ethiopia | doi: 10.1002/cam4.1334 | Outcome of interest not available |
|  | Heailemariam et al. | Magnitude and associated factors of VIA positive test results for cervical cancer screening among refugee women aged 25-49 years in North Ethiopia | doi: 10.1186/s12885-020-07344-9 | Outcome of interest not available |
|  | Aergaw et al. | Knowledge, and practice of cervical cancer prevention and associated factors among commercial sex workers in Shashemene Town, West Arsi, Oromia Region, Ethiopia | doi: 10.1186/s12905-022-01819-6 | Outcome of interest not available |
|  | Gerebregzabiher et al. | Correlates of cervical cancer screening uptake among female under graduate students of Aksum University, College of Health Sciences, Tigray, Ethiopia | doi: 10.1186/s13104-019-4570-z | Outcome of interest not available |
|  | Aoegale et al. | Knowledge, attitude and practice of cervical cancer screening among women infected with HIV in Africa: Systematic review and meta-analysis | doi: 10.1371/journal.pone.0249960 | Review article |
|  | Maerrgga et al. | Clients' satisfaction with cervical cancer screening services and influencing factors at public health facilities in Debre Markos town, Northwest Ethiopia, 2022/23: a convergent parallel mixed method | doi: 10.1186/s12905-024-03250-5 | Outcome of interest not available |
|  | Taeaklehaimanot et al. | Precancerous lesion determinants in women attending cervical cancer screening at public health facilities in North Shoa Zone, Amhara, Ethiopia: an unmatched case-control study | doi: 10.1186/s12905-024-03113-z | Outcome of interest not available |
|  | Aendrae et al | Screening and cervical cancer cure: population based cohort study | https://doi.org/10.1136/bmj.e900 | Outcome of interest not available |
|  | BT, Girma et al. | Cervical cancer screening practices and its associated factors among females of reproductive age in Durame town, Southern Ethiopia | https://doi.org/10.1371/journal.pone.0279870 | Outcome of interest not available |
|  | A. Ayenew, et al. | Uptake of cervical cancer screening service and associated factors among age-eligible women in Ethiopia: systematic review and meta-analysis | https://doi.org/10.1186/s13027-020-00334-3 | Review article |
|  | Tgsfay et al. | Evaluation of cervical cancer screening program in Gondar city administration public health facilities, Northwest Ethiopia, 2021: mixed method approach | doi: 10.1186/s12885-023-11533-7. | Outcome of interest not available |
|  | Paeirson, L.,et al | Screening for cervical cancer: a systematic review and meta-analysis | https://doi.org/10.1186/2046-4053-2-35 | Review article |
|  | Messay et al. | Health Seeking Behavior of Patients Diagnosed with Cervical Cancer in Addis Ababa, Ethiopia | doi: 10.4314/ejhs.v28i2.2 | Outcome of interest not available |
|  | Mellaku et al. | Utilization of cervical cancer screening service among female health workforces in public health institutions in south east Ethiopia, a cross-sectional study | doi: 10.1016/j.heliyon.2023.e23086 | Outcome of interest not available |
|  | Cherinet et al. | Knowledge, practice of cervical cancer screening and associated factors among women police members of Addis Ababa police commission Ethiopia | doi: 10.1186/s12885-023-11478-x | Outcome of interest not available |
|  | Wonde et al. | Burden of Cancer and Utilization of Local Surgical Treatment Services in Rural Hospitals of Ethiopia: A Retrospective Assessment from 2014 to 2019 | doi: 10.1093/oncolo/oyac127 | Outcome of interest not available |
|  | B chaka et al. | A survey of knowledge and attitudes relating to cervical and breast cancer among women in Ethiopia | doi: 10.1186/s12889-018-5958-8 | Outcome of interest not available |
|  | Ararsa, T., et al. | Knowledge towards cervical cancer screening and associated factors among urban health extension workers at Addis Ababa, Ethiopia: facility based cross-sectional survey | https://doi.org/10.1186/s12885-021-07952-z | Outcome of interest not available |
|  | AGebremariam et al. | Breast and cervical cancer patients’ experience in Addis Ababa city, Ethiopia: a follow-up study protocol | https://doi.org/10.1136/bmjopen-2018-027034 | Protocol |
|  | Berbie et al. | Cervical cancer in Ethiopia: a review of the literature | https://doi: 10.1007/s10552-022-01638-y | Review |
|  | Gerbie et al. | Human papillomavirus in Ethiopia | doi: 10.1007/s13337-019-00527-4 | Outcome of interest not available |
|  | Lakwoya et al. | Precancerous cervical lesion screening acceptance among women in Eastern Ethiopia | doi: 10.1136/bmjopen-2023-073721 | Outcome of interest not available |
|  | Gimer et al. | Cervical cancer screening uptake in Sub-Saharan Africa: a systematic review and meta-analysis | doi: 10.1016/j.puhe.2021.04.014. | Review article |
|  | Betachew et al. | Cervical cancer screening knowledge and barriers among women in Addis Ababa, Ethiopia | doi: 10.1371/journal.pone.0216522. eCollection 2019. | Outcome of interest not available |
|  | Buddies et al. | Cervical cancer screening in rural Ethiopia: a cross- sectional knowledge, attitude and practice study | doi: 10.1186/s12885-020-07060-4. | Outcome of interest not available |
|  | Destaye et al. | Cervical cancer screening utilization and predictors among eligible women in Ethiopia: A systematic review and meta-analysis | doi: 10.1371/journal.pone.0259339. | Review article |
|  | S. Shekhar et al. | Cervical cancer screening  Current knowledge & practice among women in a rural population of Kerala | https://journals.lww.com/ijmr/fulltext/2012/36020/cervical_cancer_screening__current_knowledge__.5.aspx | Outcome of interest not available |
|  | Andrae et al | Screening and cervical cancer cure: population based cohort study | https://doi.org/10.1136/bmj.e900 | Outcome of interest not available |
|  | Heena et al. | Knowledge, Attitudes, and Practices towards Cervical Cancer and Screening amongst Female Healthcare Professionals: A Cross-Sectional Study | https://doi.org/10.1155/2019/5423130 | Outcome of interest not available |
|  | Sylivia et al. | Knowledge, Attitudes, and Demographic Factors Influencing Cervical Cancer Screening Behavior of Zimbabwean Women | https://doi.org/10.1089/jwh.2010.2062 | Outcome of interest not available |
|  | Portnoy et al. | Projections of human papillomavirus (HPV) vaccination impact in Ethiopia, India, Nigeria and Pakistan: a comparative modelling study | https://doi.org/10.1136/bmjgh-2021-006940 | Outcome of interest not available |
|  | Ukomo et al. | Acceptance of Human Papillomavirus Vaccination and Associated Factors among Girls in Arba Minch Town, Southern Ethiopia, 2020 | https://doi.org/10.1155/2022/7303801 | Outcome of interest not available |
|  | YM larebo et al. | Awareness, Acceptance, and Associated Factors of Human Papillomavirus Vaccine among Parents of Daughters in Hadiya Zone, Southern Ethiopia: A Cross-Sectional Study |  | Outcome of interest not available |
|  | Thapa et al. | Cervical Cancer Screening Seeking Behavior among Female  Community Health Volunteers of Surkhet District | https://doi.org/10.1101/2024.07.25.24311012 | Outcome of interest not available |
|  | YA Ziyad et al. | Determinants of Cervical Cancer Screening among Female Health Professionals in Harar Town, Eastern Ethiopia: A Cross-Sectional Study | https://doi.org/10.1155/2024/1430978 | Outcome of interest not available |
|  | K. Tesfaw et al | Utilisation of cervical cancer screening and factors associated with screening utilisation among women aged 30–49 years in Mertule Mariam Town, East Gojjam Zone, Ethiopia, in 2021: a cross-sectional survey | https://doi.org/10.1136/bmjopen-2022-067229 | Outcome of interest not available |
|  | Kussia et al. | Health care seeking behaviour towards cervical cancer screening among women aged 30–49 years in Arbaminch town, Southern Ethiopia, 2023 | https://doi.org/10.1186/s12885-023-11810-5 | Outcome of interest not available |
|  | Beyene et al. | Human papillomavirus vaccination uptake and its associated factors among adolescent school girls in Ambo town, Oromia region, Ethiopia, 2020 | https://doi.org/10.1371/journal.pone.0271237 | Outcome of interest not available |
|  | Mivundura et al. | Evaluating the cost and operational context for national human papillomavirus (HPV) vaccine delivery in three regions of Ethiopia | https://doi.org/10.1371/journal.pgph.0003357 | Outcome of interest not available |
|  | Kassa HN, et al. | Practice and Associated Factors of Human Papillomavirus Vaccination Among Primary School Students in Minjar-Shenkora District, North Shoa Zone, Amhara Regional State, Ethiopia, 2020 | https://doi.org/10.2147/CMAR.S324078 | Outcome of interest not available |
|  | M. Bittew et al | Parental willingness to vaccinate their daughters against human papilloma virus and its associated factors in Woldia town, Northeast Ethiopia | https://doi.org/10.3389/fgwh.2024.1243280 | Outcome of interest not available |
|  | A. Zewdie et al. | Human papillomavirus vaccine acceptance among adolescent girls in Ethiopia: a systematic review and meta-analysis | https://doi.org/10.1186/s12889-023-16305-3 | Review article |
|  | Krma et al | Human papillomavirus vaccination and parents’ willingness to vaccinate their adolescents in Ethiopia | https://doi.org/10.1186/s13027-023-00535-6 | Outcome of interest not available |
|  | Woldehawariyat et al. | Uptake of human papillomavirus vaccination and its associated factors among adolescents in Gambella town, Southwest, Ethiopia: a community-based cross-sectional study | https://doi.org/10.1136/bmjopen-2022-068441 | Outcome of interest not available |
|  | Derbie, A., et al | Acceptance of human papillomavirus vaccination and parents’ willingness to vaccinate their adolescents in Ethiopia: a systematic review and meta-analysis | https://doi.org/10.1186/s13027-023-00535-6 | Review article |
|  | Tesfaye G, et al. | Human papillomavirus related cervical cancer and anticipated vaccination challenges in Ethiopia | https://pmc.ncbi.nlm.nih.gov/articles/PMC4791164/ | Outcome of interest not available |
