# Supplementary material for: Determinants of cervical cancer screening intention among reproductive age women in Ethiopia: A systematic review and meta-analysis
Source: PLoS One. 2024 Oct 31;19(10):e0312449. doi: 10.1371/journal.pone.0312449 (PMC11527304; doi:10.1371/journal.pone.0312449)
Supplement: S4 Table — (DOCX) [file pone.0312449.s004.docx]

**S4 Table: Quality assessment of included studies**

| **Studies** | **Quality assessment criteria** | | | |
| --- | --- | --- | --- | --- |
|  | **Selection** | **Comparability** | **Outcome** | **Overall quality** |
| Wollancho W et al | **** | * | ** | 7 |
| Eshetu, HB et al | **** | * | ** | 7 |
| Bishaw G et al | **** | ** | ** | 8 |
| Desta AA et al | **** | * | ** | 7 |
| Tomas G et al | **** | ** | ** | 8 |
| Meried E et al | **** | ** | ** | 9 |
| Alemnew A et al | **** | ** | ** | 8 |
| Berhe S et al | **** | ** | ** | 8 |
| Belete et al | **** | ** | ** | 8 |

The Newcastle Ottawa Scale (NOS) was used **t**o assess the quality of included articles

** Two points, *** Three points; and **** four point
